# NaFeNb(PO_4_)_3_ as an Electrode Material for Sodium-Ion Batteries: Insights into Phase Evolution and Capacity Fading

**DOI:** 10.1021/acs.chemmater.5c01854

**Published:** 2026-01-08

**Authors:** Nicolò Pianta, Shahid Khalid, Ivan Claudio Pellini, Domenico Antonio Florenzano, Gabriele Brugnetti, Nicole Ceribelli, Luca Olivi, Giuliana Aquilanti, Denis Sheptyakov, Andrew Nicholas Fitch, Martina Fracchia, Livia Giordano, Riccardo Ruffo, Chiara Ferrara

**Affiliations:** † Department of Materials Science, 9305 Università degli Studi di Milano-Bicocca, Via Cozzi 55, Milano 20125, Italy; ‡ Ricerca sul Sistema Energetico - RSE S.p.A., Via R. Rubattino 54, Milano 20134, Italy; § Elettra − Sincrotrone Trieste, Strada Statale Strada Statale 14 - Km 163,5 in AREA Science Park Basovizza, Trieste 34149, Italy; ∥ Laboratory for Neutron Scattering and Imaging, Paul Scherrer Institut, Villigen PSI 5232, Switzerland; ⊥ European Synchrotron Radiation Facility, 71 Avenue des Martyrs, Grenoble 38000, France; # Department of Chemistry, 230170 Università di Pavia, Via T. Taramelli 12, Pavia 27100, Italy; ∇ GISEL - INSTM, Consorzio Interuniversitario per la Scienza e Tecnologia dei Materiali, Via G. Giusti 9, Firenze 50121, Italy

## Abstract

The NaFeNb­(PO_4_)_3_, NFNP, material has been designed as a candidate anode material for sodium-ion batteries, as in its pristine form it combines the presence of Fe­(III) and Nb­(V)—available for possible reduction upon Na insertion—allowing for the formal introduction of 3 Na ions at reasonable potentials, and the robust NASICON structure with open channels for Na migration. The NFNP material has been successfully obtained by the solid-state route and fully characterized in terms of structure and transport properties by means of diffraction, XAS, and DFT analysis. Although promising, the electrochemical testing reveals that the initially satisfactory results in terms of capacity and Coulombic efficiencies fade upon cycling. The in-depth operando investigation, with the implementation of in situ XRD and XAS, unveiled a phase transition upon cycling; this involves the formation and accumulation of a low-symmetry secondary phase delivering lower capacity related to the Nb redox couples.

## Graphical abstract

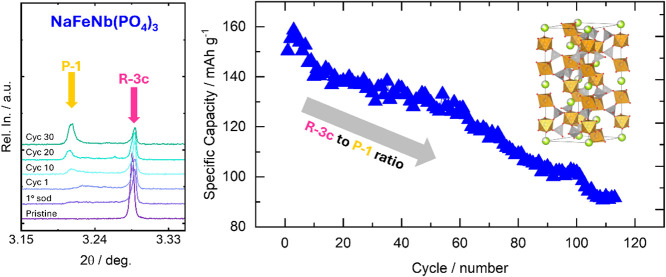

## 1 Introduction

NASICON, a Na superionic conductor, is a class of materials designed with a structure supporting high Na mobility, initially developed to create solid electrolytes.^1,2^ Since their introduction in 1976, they have been explored as Na-based conductors, but soon the focus also shifted to Li-based NASICON, with the most representative and famous composition Li_1.3_Al_0.3_Ti_1.7_(PO_4_)_3_ generally referred to as LATP and derivative compounds.^3,4^ Although initially developed as electrolytes, NASICON materials soon found applications as electrodes due to their ability to accommodate changes in the oxidation state of transition metal cations in conjunction with the insertion or extraction of alkali ions.^5^ Moreover, the possibility of varying both the type of transition metal and the alkali ion content enables the development of materials suitable for both positive and negative electrodes. The NASICON structure corresponds to the general formula AMM’(TO_4_)_3_ with A = Li, Na, K; M and M’ = transition metal(s); and T = P, Si, or both. The structure is composed of corner-sharing [M/M’O_6_] and [TO_4_] units forming a 3D stable scaffold, and a 3D percolating network is obtained where suitable voids for the accommodation and motion of A elements are found. The structure is extremely robust thanks to the presence of the polyanionic framework; the tuning of the M, M’ composition has led to the development of a plethora of electrolyte, cathode, and anode materials.^6–9^ The peculiar features that make NASICON appealing are the combination of a robust yet open polyanionic framework, tunable bandgap and operating voltage, and good ionic conductivity at room temperature (in the range of 10^–5^–10^–3^ S cm^–1^). At the same time, some drawbacks must be mentioned such as the requirement of high temperature solid-state synthesis, the formation of secondary phases, difficult to eliminate or minimize, the high interfacial resistance, and the polymorphism. When NASICON electrode materials are considered, specific challenges must be faced: the poor electronic conductivity/slow electrode kinetics, the operating voltage, the phase transitions induced by charge/discharge processes.^10–12^ These challenges are interconnected: the low electronic conductivity is intrinsic to such polyanionic materials (not only NASICON-based) and can be overcome with the introduction of carbon coating and/or blending with conductive media, combined with particle size reduction.^13–15^ The operating voltage is tuned through the definition of the composition on the M sites: cathodes have been reported exploiting Fe, Mn, Cu, Ti, Zr, and V metal centers, often with mixed compositions, involving also the high-entropy approach.^9,10,16,17^ A similar approach has also been explored for the development of negative electrodes, exploiting mainly V, Nb, Ti, Zr, and Cr elements, but with significantly fewer reported compositions.^10,18–21^ Of the various compositions, the most famous is undoubtedly NaTi_2_(PO_4_)_3_ (NTP), which is known to provide potentially high capacity and good cycling stability.^22^ Much of the literature focuses on tailoring its microstructure—through strategies such as carbon coatings, nanotube/graphene composites, oxygen-vacancy engineering, or novel morphologies like hollow particles, core–shell systems, and single crystals—to enhance cycle life and performance under high current densities.^22–28^ The main drawback of NTP, however, is its operating potential (ca. 2.1 V vs Na^+^/Na), which is relatively high for an anode material, especially in devices where stable organic electrolytes or highly concentrated water-based electrolytes are considered, and where a lower-potential anode could be exploited.^29,30^

Recently, niobium-based NASICONs have been proposed by Patra et al.,^31^ who synthesized NaNb_2_(PO_4_)_3_, a material able to store up to 150 mAh g^–1^ of reversible capacity, with an operative potential of 1.46 V vs Na^+^/Na. Despite this, such a material showed a series of phase transitions during the intercalation process, which resulted in a rather low capacity retention (60.2% after 200 cycles at 1C). The same group addressed this problem by synthesizing Na_1.5_V_0.5_Nb_1.5_(PO_4_)_3_, and later NaV_0.25_Al_0.25_Nb_1.5_(PO_4_)_3_, compounds which showed similar capacities and operative potentials but significantly higher capacity retention upon cycling, mainly due to the different reaction processes: from a series of phase transitions to a simpler biphasic reaction.^32,33^

Other authors have reported Nb-based NASICONs or NASICON-derived anodes, with compositions containing Al,^34^ V,^20,35,36^ Ti,^35^ Cr,^21^ or NbO_2_,^19^ all with the aim of increasing the specific capacity or the stability upon cycling.^33^ NASICON chemistry, which is easily tunable, is indeed the main strength of this class of materials, especially in systems where a very low anode potential can be detrimental (like aqueous-based systems) or where very high currents are required. At the same time, the specific element or elemental compositions on the M/M’ site and their oxidation states determine the starting occupancy on the A sites (i.e., the Li/Na content and thus also the theoretical capacity) and, as a consequence, the initial structure. Indeed, it is well known that the NASICON materials can assume different structures with stability related to the (M–O)/(T–O) ratio and the A occupancies, as recently rationalized.^6^ Moreover, the structure evolves with the charge/discharge as the phase transitions are related to the A content.^6,37^ The problem is exacerbated with Na compared to the Li case due to the larger ionic radius of the former. As mentioned, the structure evolves with the A extraction and insertion; the NASICON framework provides a robust open framework usually able to retain the original symmetry for significant variation in the A content. In addition to this, a wide variety of NASICON compositions can sustain reversible phase transitions upon cycling, with the most frequently reported among R-3c and C2/c structures; far fewer reports have appeared on irreversible phase transitions to lower symmetry systems.

With the aim of developing and optimizing improved electrode materials for use in traditional ion-intercalation batteries or emerging aqueous or hybrid aqueous/organic chemistries, it is essential to gain a deep understanding of the phase transformations that may occur during electrochemical processes in NASICON phases. For this reason, we explore here the composition NaFeNb­(PO_4_)_3_, NFNP, considered as a candidate for rechargeable battery anode material. Indeed, the presence of Fe­(III) and Nb­(V) as an initial hypothesis, involves the possibility of exploiting the Fe­(III/II), Nb­(V/IV), and Nb­(IV/III) couples with the insertion of 3 Na ions within the structure, leading to a theoretical capacity of 176.07 mAh g^–1^ with expected operating potential compatible with organic and aqueous electrolytes. Also, to our knowledge, Fe has not yet been explored in Nb-based NASICON anodes for sodium-ion batteries. The NFNP material has been deeply characterized in terms of initial structure, Na content, sites, and diffusion paths through the implementation of X-ray diffraction, temperature-dependent neutron diffraction, XAS, and DFT investigation. The material has then been tested in half-cell configuration vs Na, demonstrating good initial capacity but a fading of performance with cycling. An in-depth analysis of the NFNP under working conditions through the implementation of operando XRD and XAS analysis revealed a noncompletely reversible phase transition, identified as the origin of the loss of capacity.

## 2 Methods

### 2.1 Synthesis and Carbon Coating

NaFeNb­(PO_4_)_3_ was synthesized via a conventional solid-state approach. In particular, Nb_2_O_5_ (Alfa Aesar, 99.5%), (NH_4_)­H_2_PO_4_ (Fluka, >99%), Na_2_CO_3_ (Sigma-Aldrich, 99.95%), and FeC_2_O_4_ (Sigma-Aldrich, 99%) were mixed in stoichiometric amounts using a hand mortar. The solid-state synthesis procedure was optimized with the aim of maximizing the purity and crystallinity of the final desired phase. The temperature of the final heating treatment was varied between 900 and 1200 °C, and the holding time was explored between 12 and 72 h. The best results were obtained by treating the powders in alumina crucibles with the following steps: 300 °C for 12 h (heat ramp 1 °C min^–1^), 600 °C for 5 h (heat ramp 5 °C min^–1^), and 950 °C for 72 h (heat ramp 5 °C min^–1^). Between each step, the powders were ground again with a mortar.

To improve NASICON’s electronic conductivity, the pristine powders were coated with carbon. A portion of the powders was mixed with d-glucose (Sigma-Aldrich, >99.5%) keeping the ratio between carbon and NASICON at 10 wt % and then treated at 650 °C for 2 h under a controlled Ar atmosphere. Hereafter, the NaFeNb­(PO_4_)_3_ uncoated, pristine material will be referred to as “NFNP_uncoated,” while the material obtained after carbon coating will be labeled “NFNP”.

### 2.2 Structural, Morphological, and Physicochemical Characterizations

XRD analyses were performed to evaluate the success of the synthesis. Patterns were acquired with a Rigaku Miniflex 600 diffractometer using copper Kα as the radiation source. Measurements were carried out in the angular range 10°–70° with a 0.02° step and a scan rate of 1° min^‑1^. The obtained diffraction data were analyzed according to the Rietveld method using the FullProf suite .^38^ Neutron powder diffraction data were acquired at the HRPT beamline at the Swiss Spallation Neutron Source (SINQ) in PSI.^39^ Powders were loaded into vanadium sample holders. Measurements were performed in the 25–700 °C range, exploiting the furnace, using the high-intensity mode of the instrument and a wavelength of λ=1.494 Å in the 4°–165° angular range, with a step size of 0.05°. Crystal structure models were verified with Rietveld refinements, using the FullProf suite, which was also used for BVEL calculations.^38^ Morphological analysis was performed with an SEM Zeiss Gemini electron microscope. To avoid charging effects during measurements, the samples were preliminarily metallized with graphite. The carbon-coated sample was also analyzed with a JEOL JEM 2100 Plus (TEM) instrument operated at 200 kV in imaging mode. To evaluate the amount of carbon present on the sample after coating, thermogravimetric analysis (TGA) was performed using a Mettler Toledo TGA/DSC-1 instrument with a heating rate of 5 °C min^–1^ in the temperature range 30–1000 °C. The TGA analysis was performed on both pristine and coated samples, and the percentage of carbon due to the coating was evaluated as the difference between the residual masses of the two samples at 600 °C.

The ionic conductivity of the NASICON was measured through electrochemical impedance spectroscopy. In particular, a cylindrical pellet (diameter: 1.2 cm, width: 0.1 cm) was prepared using an oil press (pressure: 10 tons) and then synthesized at 950 °C for 24 h to increase the relative density (ratio between the density of the pellet and that of the NASICON, as calculated from Rietveld refinement). The two faces of the pellet were coated with gold to improve the electrical contact between the ionic conductor (NASICON) and the external circuit. Impedance was measured in a homemade high-temperature probe from 350 to 700 °C. Electrode formulations were prepared by mixing active material, conductive carbon (Super-P), and binder (PVDF) in an 8:1:1 mass ratio in n-methyl-2-pyrrolidone; an Ika Ultra-Turrax T10 disperser was used for this purpose. For Na cell assembly, the electrodes were fabricated using the so-obtained slurries cast onto an aluminum foil with a thickness of 20 μm, dried at 80 °C under vacuum, and then pressed with a calender (active mass load is 1.27 mg cm^–2^).

### 2.3 Electrical and Electrochemical Characterizations

Electrochemical tests were carried out using a BioLogic VSP-300 potentiostat/galvanostat. Hohsen CR2032 coin cells were assembled to test the active material formulation as the working electrode. Metallic Na was used as the counter electrode. A glass microfiber (Whatman), soaked with a 1 M solution of NaClO_4_ in propylene carbonate (PC) with 2% fluoroethylene carbonate (FEC), was used as the electrolyte/separator. Rate tests were conducted by reducing and oxidizing the NFNP with a constant current of different values. A typical rate test consisted of imposing currents of 23, 117, 234, 468, and 23 mA g^–1^ for 10 cycles each, from 3 to 0.75 V vs Na^+^/Na. To obtain detailed information from differential capacity, a potentiodynamic cycling with galvanostatic acceleration (PCGA) was used. It consists of small potential steps (5 mV), which are maintained until the current drops below a certain limit (|I| < 2.34 mA g^–1^) to allow for a complete reaction with small overpotentials.

### 2.4 Operando Analysis

X-ray absorption spectra were acquired at the Nb and Fe K-edges at the XAFS beamline at the Elettra synchrotron radiation facility in Trieste, Italy. Ex situ spectra of reference samples (NbO_2_, Nb_2_O_5_, FeO, Fe_3_O_4_, and α-FeOOH) were measured in transmission after pelletizing a proper amount of each sample, so as to have optimized absorption, together with cellulose. The spectrum of NbO was kindly provided by Prof. Sanjeev Mukerjee.^40^ For the operando experiment, XAS spectra were acquired in fluorescence mode using a silicon drift detector. Due to the different experimental conditions required to acquire XAS spectra at each edge, the experiments could not be conducted simultaneously. However, the measurements at each edge were carried out under identical conditions to ensure a reliable comparison of the results. The ring current and energy were 200 mA and 2.4 GeV, respectively. A Si(311) for the Nb K-edge, and Si(111) for the Fe K-edge double crystal monochromator, was used to ensure high-order harmonic rejection by detuning the second crystal. A water-cooled, Pt-coated silicon mirror was used to obtain the vertical collimation of the beam. XAS spectra were acquired during the charge–discharge process of the cell, each spectrum lasting approximately 1 h. The operando XAS measurements were performed by oxidizing and reducing NFNP by collecting XANES spectra at the Nb K-edge, and then, the experiment was repeated at the Fe K-edge, fluorescence mode). To do so, an ECC-Opto-Std cell (El-cell) equipped with a polyimide window was used. The cell was assembled similarly to what was already described for the sodium-based coin cells, with the main difference in the current collector for the working electrode, which in this case was carbon cloth, to avoid the attenuation of X-rays by Al. The working electrode was cycled once between OCV and 0.8 V vs Na^+^/Na, with a current of 15 mA g^–1^. For selected and relevant points during the cycling, electrodes were recovered from cycled coin cells and examined in ex situ mode to check the reproducibility of the data and validate the operando experiment. The X-ray signal extraction and analysis was performed by means of Athena, belonging to the set of interactive programs IFEFFIT.^41^ For the X-ray absorption near edge structure (XANES), the spectra were first background-subtracted using a straight line and then normalized to unit absorption above 500 eV after the absorption edge. Operando XRD measurements have been collected at ID22 beamline at ESRF.^42^ Data were collected by oxidizing and reducing NFNP while collecting XRD patterns in high resolution mode, at a wavelength of 0.35429(1) A, scanning the 13-crystal multianalyzer stage at 5°/min from −10° to 40°. Data were corrected for axial divergence, and the 13 channels combined in steps of 0.002°. A homemade cell equipped with two beryllium windows was used to perform the in situ XRD investigation. A self-standing working electrode was used to allow for X-ray penetration. Such an electrode was prepared by mixing the active material with conductive carbon and a suspension of PTFE in water as binder (active material: carbon: binder ratio 8:1:1). The resulting mixture, similar in consistency to a dough, was calendared several times until a self-standing foil (thickness: 120 μm) was obtained. The electrode was then dried at 80 °C overnight before cell preparation. The choice of current collector—carbon cloth for operando XAS analysis and a self-standing electrode for operando XRD—was dictated by the design of the respective cells. In the ECC-Opto-Std cell used for XAS, the window is insulating and electrical contact is provided by a single pin. As a result, the electrode itself must be sufficiently conductive to ensure an efficient electron flow and uniform material reactivity. In contrast, the operando XRD cell uses a conductive window, allowing the electrode to draw electrons directly from it. This eliminates the need for a separate current collector, thereby minimizing the amount of material that could interfere with the X-ray measurements.

### 2.5 Calculations

Vienna Ab Initio Simulation Package (VASP)^43–45^ was used to perform Density Functional Theory (DFT) calculations employing the generalized gradient approximation of the exchange and correlation functional with the Perdew–Burke–Ernzerhof (PBE) formalism,^46^ Projector Augmented Wave (PAW)^47^ for the description of the core–electron interaction, and Grimme’s D3 parametrization for the dispersion forces.^48^ The correct description of electron localization with the DFT-based method requires the correction of self-interaction, usually obtained with DFT + U or hybrid functional methods. In this project, we use the DFT + U approach,^49,50^ where a U_eff_ = U–J is added on the transition metal d states of the first-row transition metal, with Ueff optimized by fitting the formation enthalpies of oxides.^51,52^ Here, the Hubbard-optimized parameter for the d-orbitals of Fe is set to U – J = 5.3 eV. Brillouin zone integration is performed using the Monkhorst–Pack method,^53^ employing a (3 × 3 × 1) k-point grid. The plane-wave basis set is employed with a cutoff energy of 400 eV. The threshold criteria for electronic and ionic loops were set to 10^–6^ eV and 10^–2^ eV/Å, respectively. All performed calculations are spin-polarized, with the initialization of a ferromagnetic spin ordering on the Fe and Nb ions. The diffusion dynamic behavior was simulated via the climbing-image nudged elastic band (CI-NEB) method.^54^ A battery based on the NASICON anode material implies the reversible insertion/extraction of Na^+^ ions into/from the Na_*x*_FeNb­(PO_4_)_3_ framework according to the redox reaction of (eq 1):

$$Na_{x}FeNb{\left(PO_{4}\right)}_{3}+\Delta x\;{\mathrm{Na}}_{\mathrm{bulk}}\to Na_{\left(x+\Delta x\right)}FeNb{\left(PO_{4}\right)}_{3}$$ 1

where *x* and (*x* + Δ*x*) are the initial and final Na concentrations in the NASICON structure. The average voltage with respect to the Na^+^/Na redox couple across an intercalation extent can be calculated from (eq 2):

$$V=-\frac{\Delta G}{\Delta x\;F}=-\frac{E_{{\mathrm{Na}}_{(x+\Delta x)}FeNb{\left({PO}_{4}\right)}_{3}}-\left[E_{{\mathrm{Na}}_{x}FeNb{\left({PO}_{4}\right)}_{3}}+\Delta x\left(E_{{\mathrm{Na}}_{bulk}}\right)\right]}{\Delta x\;F}$$ 2

## 3 Results and Discussion

### 3.1 Physicochemical Characterization of NFNP

The success of the NaFeNb­(PO_4_)_3_ synthesis was validated via XRD analysis, as reported here in Figure 1. The NFNP_uncoated pattern can be rationalized considering the R-3c space group, describing the majority of the NASICON structures.^10,11,55,56^ The fitting (Figure 1a) has been based on considering also the presence of NbPO_5_ (card no. 98-003-6626) and NaFeP_2_O_7_ (card no. 98-003-7013) as minor phases to properly account for the reflections at 15.7°, 21.5°, and 22.9° (as the most relevant), not originating from the NASICON structure. These phases have been quantified as 0.79% and 4.58% respectively thus the NFNP constitutes 94.63% of the sample. The obtained cell parameters, *a* = 8.59154(6) Å and *c* = 22.0468(2) Å, are in line with those reported for the same composition^57,58^ and generally in trend with those of such materials and the rational geometrical parameters recently defined for this system.^6^ The results obtained from room-temperature neutron and XRD data are consistent and are reported in Table S1.

**Figure 1:**
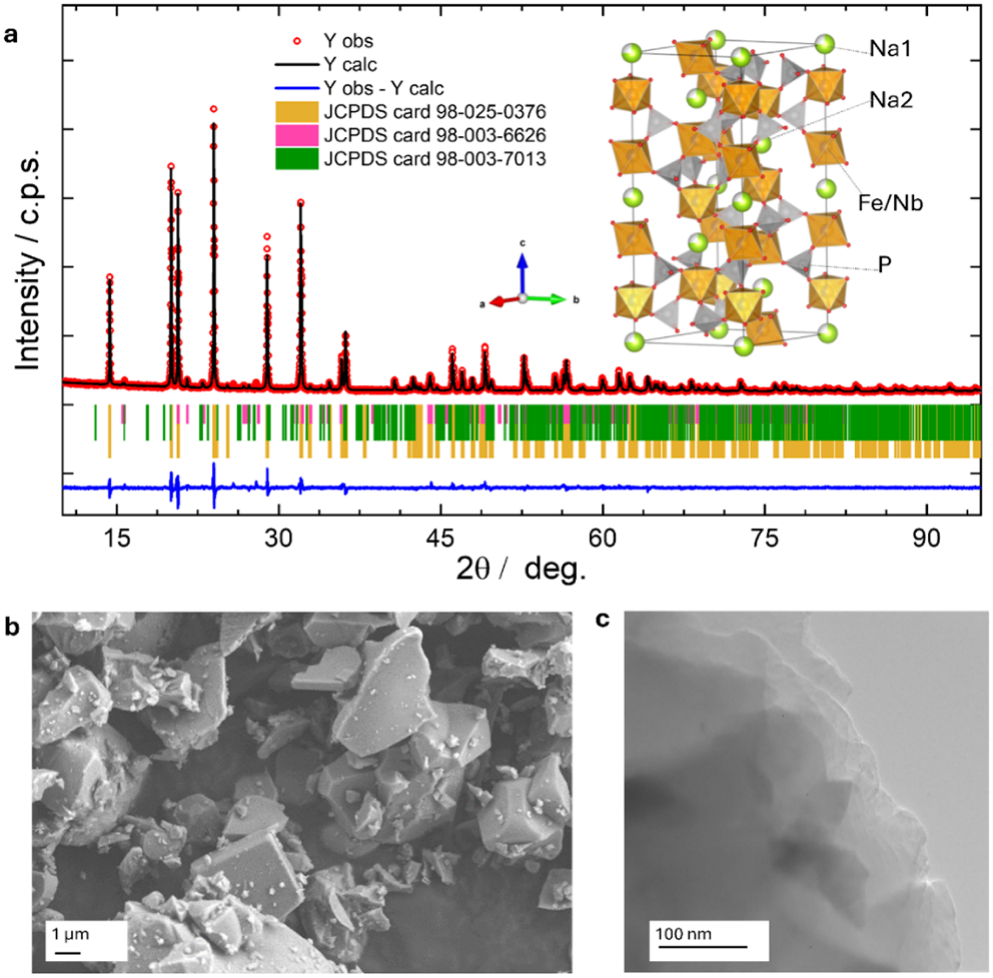
(a) XRD patterns for NFNP and results from Rietveld refinement based on the presence of the NaFeNb­(PO_4_)_3_ structure (card no. 98-025-0376) and two minor impurities: NbPO_5_ (card no. 98-003-6626) and NaFeP_2_O_7_ (card no. 98-003-7013), together with the representation of the refined structure (P polyhedral in gray, Nb/Fe polyhedral in orange, Na ion in lime green). The agreement factors for the refinements are R_wp_ = 4.44, χ^2^ 2.67; (b) SEM and (c) TEM images of the NFNP powder.

Considering the NFNP structural details, the NASICON rhombohedral structure AM­(TO_4_)_3_ is based on the presence of corner-sharing [MO_6_] and [TO_4_] units forming a 3D stable structure, with M (Fe/Nb) and T (P) occupying the 12c and 18e sites, respectively; oxygen ions are found on O1 12c (0,0, z) and 18e (x, 0, 1/4) sites. Within this framework, the Na ions have been described to occupy the A1 6b site (0,0,0) and A2 18e site (x, 0, 1/4); with the content of Na from 0 to 1, the A1 is first occupied, while for higher Na content, the A2 sites are populated up to the final stoichiometry A_4_M­(TO_4_)_3_. The A1 site is coordinated to six O2 ions, while the A2 site has lower symmetry and is coordinated to eight oxygen sites, half O1 and half O2.

As per the above-mentioned description, the Fe/Nb share the same crystallographic site; their occupancies have been refined, and the values obtained globally agree with the desired nominal composition (0.45:0.56 and 0.54:0.46 ratio determined from X-ray and neutron data, respectively; see Table S1). In the starting model, sodium was positioned on the A1 site; attempts to also introduce the second site lead to near-zero occupancy of this position, as expected and in agreement with the results from DFT calculations, confirming that the A1 site is more stable. Overall, the refined value for the Na1 occupancy indicates a lower value with respect to the nominal one (0.83 vs 1, as determined both from neutron and synchrotron data). This sodium deficiency may be considered an advantage from an electrochemical point of view since these materials were designed for use as anodes (i.e., the charging step is reduction/sodium insertion).

This model has been considered the starting point for the analysis of the temperature-dependent diffraction data and operando XRD, as discussed in the following sections.

The morphology of the obtained powder, as evidenced from the SEM images in Figure 1b, is also typical for such materials synthesized at high temperatures, presenting large, smooth, irregular, cubic-shaped crystallites of about 1–5 μm. SEM-EDX maps (Figure S1) indicate that Fe and Nb are homogeneously distributed within the sample, with no clustering effects. The carbon coating was introduced to enhance the poor electrical conductivity of the material, which is related to the peculiar distorted polyhedral interconnection^59^ and was obtained through thermal treatment with the addition of glucose, as described in the experimental section. The coating procedure does not affect either the crystal structure or the morphology of the materials, as evidenced by the comparison of XRD (Figure S2a-b) and SEM (Figure S2 c-d-e-f) data of the pristine and coated materials. The final content of carbon can be estimated to be around 6.2 wt %, as seen in the TGA analysis, from the difference between the retained masses of pristine and coated samples at 600 °C (Figure S3). Finally, the TEM analysis, reported in Figure 1c, reveals that the obtained coating is homogeneous, with an average thickness of 40 nm. The presence of the coating is evident, particularly from the analysis of the TEM images of the NFNP_uncoated and NFNP samples (Figure S2g-h-i-j). The nature of the coating has been further confirmed by CHNS analysis (Table S2) indicating a carbon content in the order of 4% for the NFNP sample, while the NFNP_uncoated powder presents negligible carbon content, in very nice agreement with the thermal analysis.

#### 3.1.1 Ionic Conductivity

The sodium ion conduction mechanisms in the pristine material were evaluated by neutron diffraction, and the results are presented in Figures 2 and 3. The neutron diffraction data were collected in the 25–700 °C range (Figure 2b) and are displayed together with the cell parameters obtained from the Rietveld refinements (Figure 2a) as a function of temperature (Figure 2c). The patterns evolve with temperature with a small shift of the peaks; no evidence of phase transition can be detected, and the R-3c structure is retained for the whole temperature range, as evident from Figure 2b.

**Figure 2:**
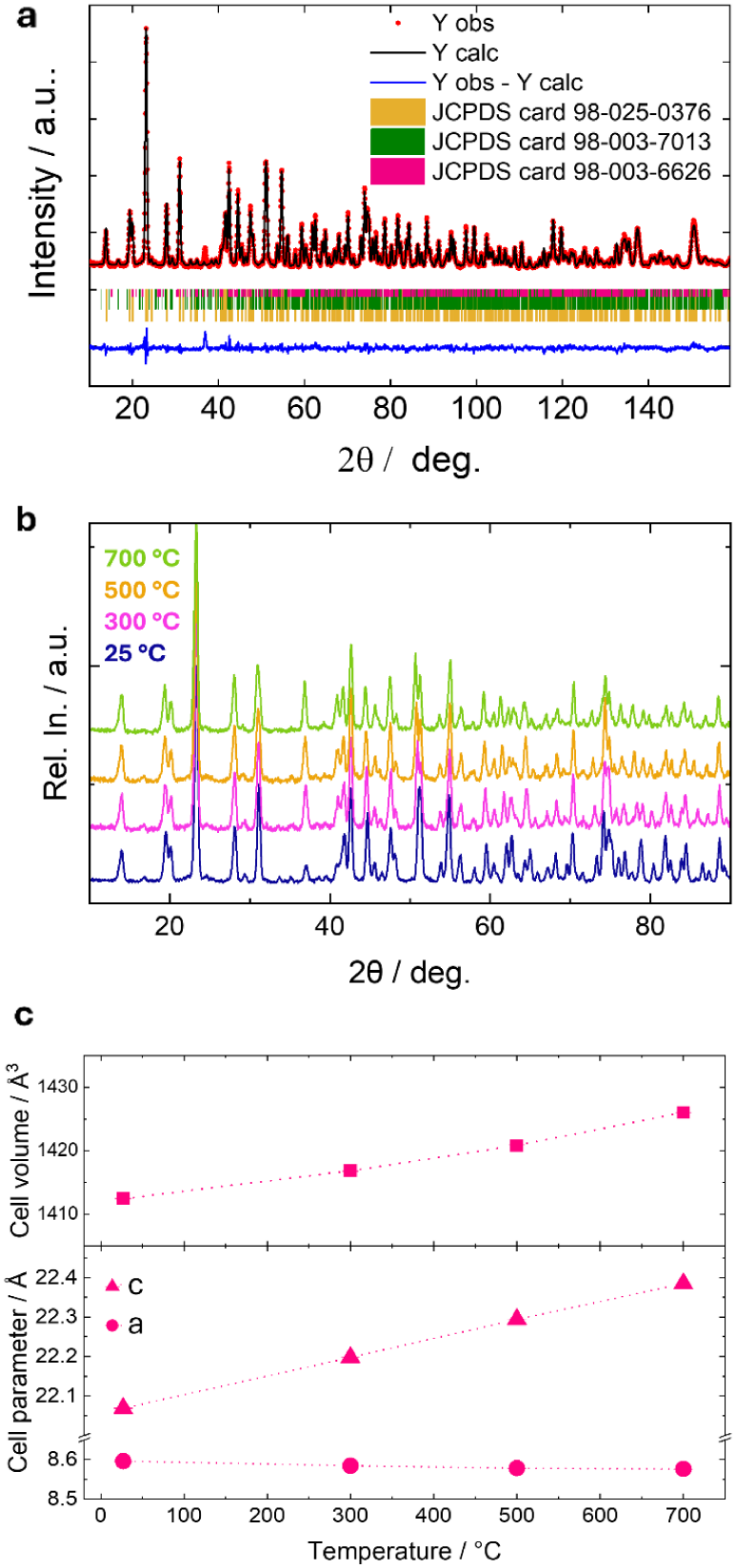
(a) Rietveld refinement of room temperature neutron data; (b) evolution of neutron data collected at 25, 300, 500, and 700 °C; (c) evolution of the cell parameters and volume as a function of temperature.

**Figure 3:**
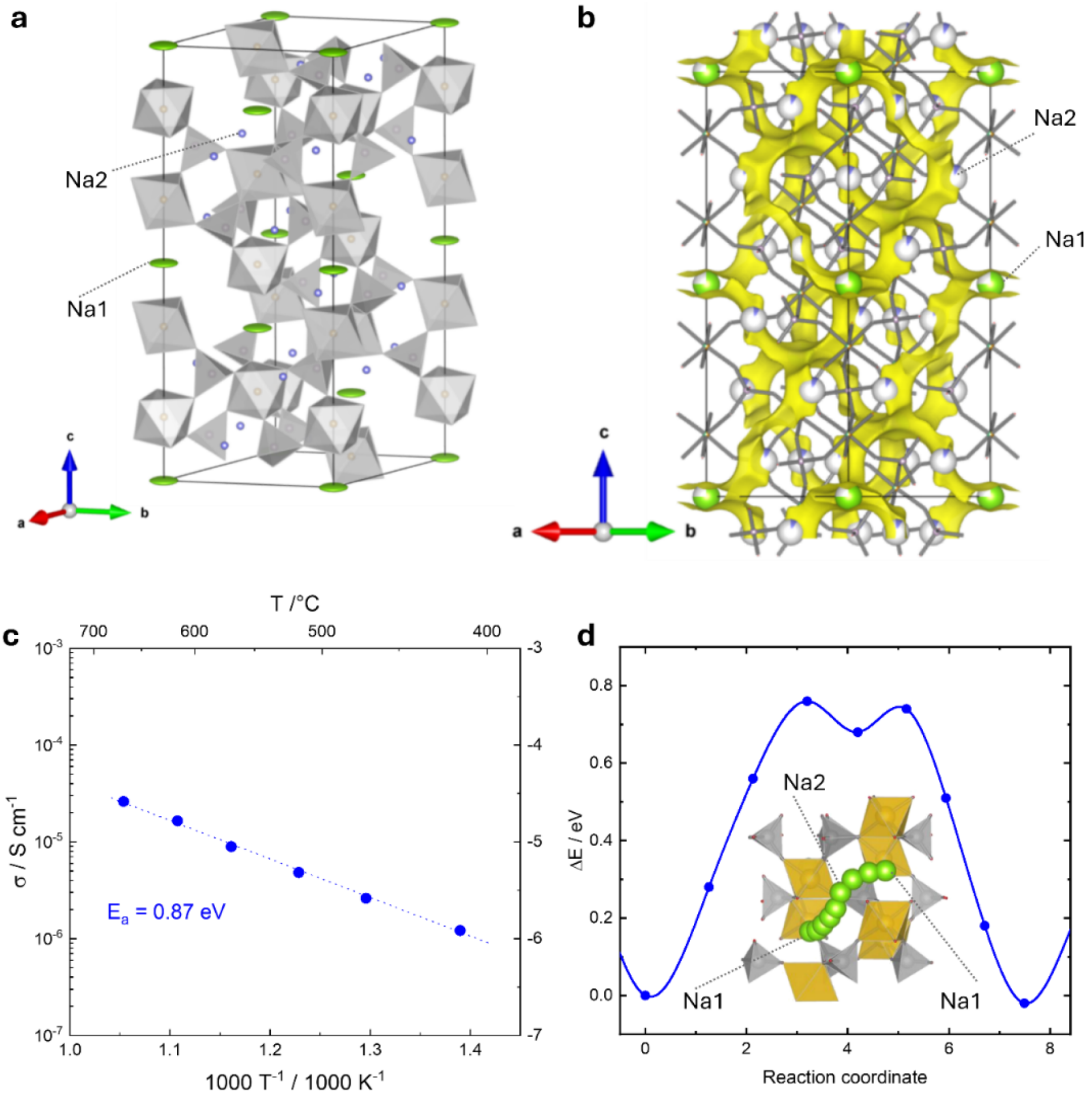
(a) Structure of NFNP and anisotropic displacement factors for Na1 sites, as determined from refinement of neutron data at 700 °C; (b) BVEL results obtained from the neutron data at 700 °C; (c) ionic conductivity of NFNP, together with its activation energy measured on a 99% dense pellet between 445 and 765 °C; and (d) diffusion path and activation energy from DFT.

The cell parameters undergo anisotropic variation with temperature, with a small contraction of the *a* parameter (8.5967(2) Å and 8.5768(4) Å at 25 and 700 °C, respectively), while the *c* parameter expands (from 22.0690(4) Å to 22.384(2) Å in the same temperature range). Overall, the cell volume variation is dominated by the *c* expansion; this volume variation is nevertheless limited (0.9% of volume variation), which is not surprising for the rigid and interconnected NASICON structure.^10,11,55,56^ Details on the results are reported in Table S3.

At the highest temperature, efforts were made to refine the anisotropic displacement factors (ADFs) for Na1, with the results presented in Figure 3a. This result supports the tendency of Na ions to diffuse within the structure via the previously proposed pathway along the Na1–Na2–Na1 channels, ultimately enabling three-dimensional diffusivity.^60–62^ Furthermore, the possibility of introducing Na ions on Na2 sites has been evaluated (Table S3); unlike the room temperature data, where the refined Na2 occupancy was negligible, the Na2 occupancy at high temperature is non-null (Na1_(0.75)_Na2_(0.08)_FeNb­(PO_4_)_3_), further suggesting the possible Na1–Na2–Na1 diffusion pathway. In order to achieve this, the refinements have been made considering isotropic displacement factors for Na species while allowing the occupancy to vary to avoid severe correlation of parameters, with a protocol similar to those reported in literature.^63,64^

To delve deeper into this aspect, the bond valence energy landscape (BVEL) method has been exploited. BVEL is an effective tool to identify the most favorable transport pathways for ion carriers and the corresponding energy barrier values in ionic solids.^65,66^ The bond valence sum (BVS) calculations show that the calculated valence states are similar to the nominal oxidation state values and results obtained from the analysis of XAS data (see discussion below), with Nb­(+5), Fe (+3.2), Na­(+1), O(−2), and P (+5), thus confirming that it is possible to consider this NASICON as an ionic compound. The results of the BVEL, reported in Figure 3b and superimposed on the crystal structure determined at the same temperature, suggest a 3D diffusion trajectory involving the movement of Na ions through the Na1 and Na2 sites, in nice agreement with the analysis obtained from temperature-dependent neutron diffraction data. The calculation has been performed at 25 and 700 °C; no variations in this path can be evidenced moving from RT to HT. The percolation mechanism is suggested to be 3D, involving the movement through the Na1–Na2–Na1 sites, as discussed; the calculated activation energy is 1.5 eV. Although this value is pretty high in comparison with the one determined from impedance spectroscopy and discussed hereafter, it must be stressed that the BVEL calculation often overestimates the barrier energies due to the limitations and simplifications of the underlying model.^65,66^

The ionic conductivity of the material (i.e., of a dense pellet of the NASICON, with a relative density of 99%) has been measured as a function of temperature, and the results are presented in Figure 3c. As expected, the conductivity follows the Arrhenius equation (i.e., linearity between the logarithm of the conductivity and the inverse of the temperature). Despite being slightly lower than that of similar materials at the same temperature,^67^ the NASICON’s ionic conductivity is in line with that of other sodium anodes,^68^ reaching 10^–5^ S cm^–1^. Also, the activation energy, with a value of 0.87 eV, is in line with that of other NASICONs.^69^

With the aim of correlating ionic conductivity data with the percolation path, DFT-based calculations have been performed. As reported in Figure 3d, the Climbing-Image Nudged Elastic Band (CI-NEB) energy profile shows two maxima for the diffusion pathway: one when moving from a Na1 site to a Na2 site, and another when moving from Na2 back to a Na1 site. This suggests a theoretically reversible diffusion path with distinct energy barriers in each direction. The first maximum reflects the energy required for Na ions to overcome the initial barrier when moving from Na1 to the local minimum Na2. After reaching Na2, the ion faces a small energy barrier to return to either the starting Na1 or the neighboring Na1 site, with a slight asymmetry in the energy profile between these sites due to the different coordination environments of specific Na1 and Na2 in the DFT model used to describe the partially occupied sites in the structure, which affects the ionic conductivity and migration efficiency.

The 3D migration vacancy-based mechanism involving the passages across A1 and A2 sites is well described for other trigonal NASICON-based compositions, and the energy barrier is related to the bottleneck size—a well-known effect in the NASICON system—related to the distorted tetrahedral site in the trigonal phase (36f site). This site can be seen as the one determined by the shared face of the 6b (A1) and 18e (A2) sites, representing the main barrier in Na diffusivity in such structure.^70–72^

The computed activation energy is of the order of 0.8 eV, fully in line with the experimental value obtained from EIS measurements and previous reports,^70^ while the percolation path determined from DFT supports the experimentally determined results from diffraction analysis.

The atom-projected DOS of NaNbFe­(PO_4_) has been calculated and coupled with the information arising from the XAS analysis; the results are reported in Figure 4. The DOS graph shows distinct regions associated with the cation and anion orbitals, separated by a moderate electronic band gap, indicating a semiconducting nature of these compounds. The DOS is displayed for spin-up and spin-down states in the range of interest around the Fermi level (Figure 4a and Figure S5 for the sodiated compositions). From the projected DOS, the valence band primarily consists of the PO_4_^3–^ electronic states contributed by the oxygen 2p orbitals, which form the highest occupied states. The conduction band is mainly made up of Nb and Fe d-orbitals, which form the lowest unoccupied states and facilitate electronic transitions when electrons are excited from the valence band, potentially contributing to the material’s semiconducting properties. The computed band gap of NaNbFe­(PO_4_)_3_ is 2.69 eV and falls within the wide range of phosphate-based NASICON materials, strongly modulated by metal composition.^5,73,74^

**Figure 4:**
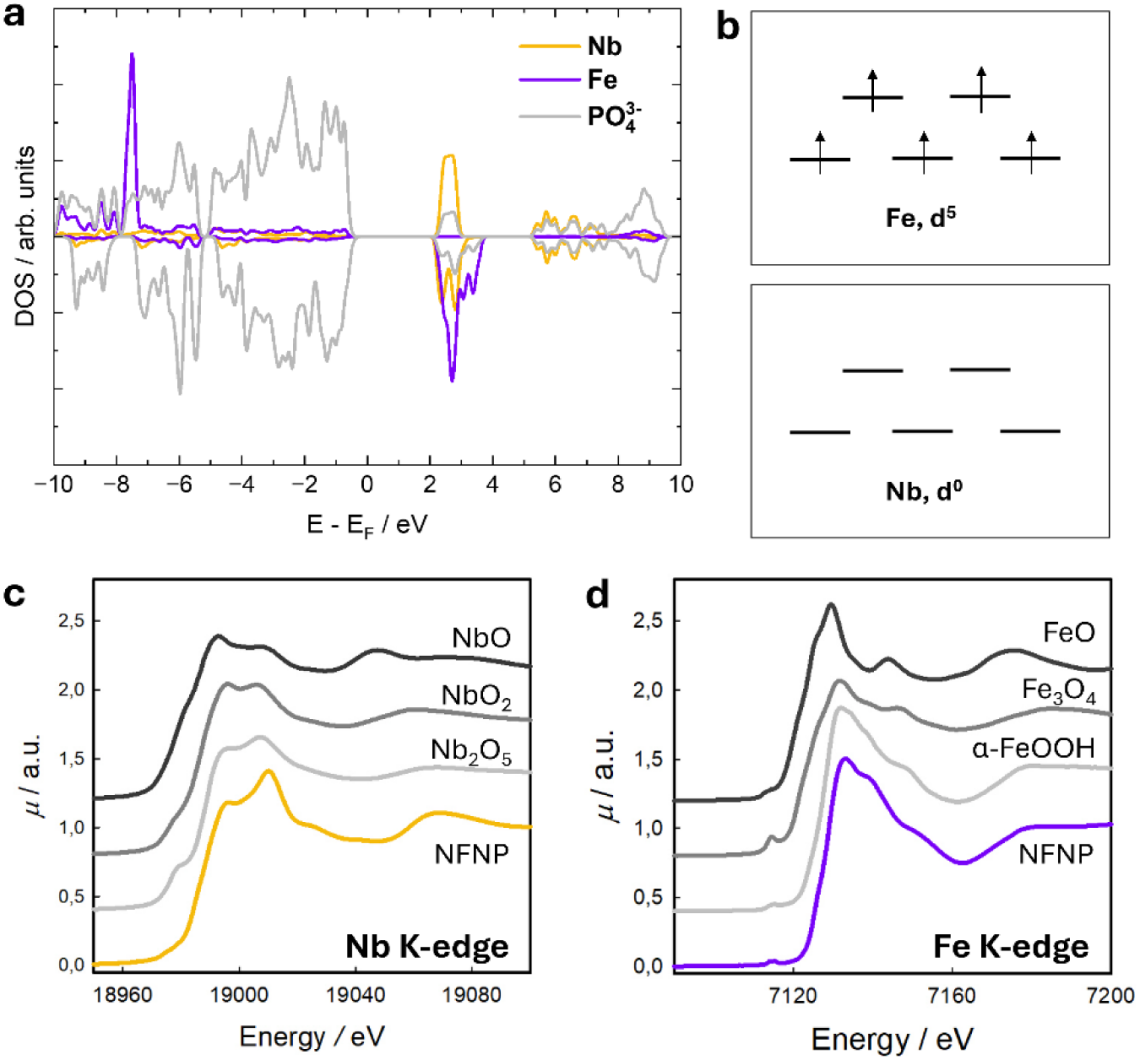
(a) Atom-projected DOS of NaNbFe­(PO_4_)_3_; (b) oxidation state; (c) Nb K-edge; and (d) Fe K-edge obtained for the NFNP sample, together with reference compounds.

In general, the oxygen 2p states of PO_4_^3–^ tend to dominate the valence band, Fe d-orbitals are dominant near the Fermi level, and Nb d-orbitals contribute more to the conduction bands. The oxidation states for the pristine NFNP composition are reported in Figure 4b, and Nb is associated with the (V) oxidation state, resulting in an electronic configuration of [Kr], with empty 4d orbitals. This high oxidation state is stabilized by the surrounding oxygen atoms in the phosphate framework. In the (III) oxidation state, Fe has an [Ar] 3d^5^ configuration. This half-filled 3d orbital configuration is relatively stable and coordinated with oxygen atoms in the structure, helping to maintain the integrity of the NASICON lattice. From the structural point of view, the relaxed structure is in line with the experimentally obtained data from diffraction analysis, with cell parameters *a* = *b* = 8.6008 Å and *c* = 21.6803 Å, obtained considering P1 symmetry. As already discussed, the stability of the two possible Na sites (Na1 and Na2) has been tested; the most favorable position for the introduction of Na, corresponding to the Na_1_FeNb­(PO_4_)_3_ stoichiometry, is the Na1 site.

This picture, which emerged from theoretical investigation, is in line with the oxidation states determined through XAS measurements. The XANES spectra of pristine NaNbFe­(PO_4_)_3_ at the Nb and Fe K-edges are shown in Figure 4c-d, in the left and right panels, respectively. Concerning the Nb K-edge, it is possible to infer that Nb is characterized by the (V) oxidation state in the starting material. Indeed, the spectrum of NFNP, compared with the spectra of NbO, NbO_2_, and Nb_2_O_5_, taken as references, shows the energy of the rising edge coincident with that of Nb_2_O_5_, indicating that the oxidation state in the two compounds is the same. The spectral shape is in turn similar to the empty NASICON Nb_2_(PO_4_)_3_ previously reported in the literature.^31^ Concerning the spectrum at the Fe K-edge, the edge energy position is coincident with that of α-FeOOH (goethite), indicating the presence of Fe­(III) in the pristine sample. It must be noted that the spectrum profiles are also similar. Indeed, goethite is composed of edge-sharing Fe­(III)­O_6_ octahedra,^75^ leading to a similar Fe local structure as in the NASICON. Therefore, the XAS data confirm that the oxidation states of Fe and Nb coincide with the nominal ones expected by the stoichiometry.

Overall, the picture emerging from the physicochemical characterization of the NFNP sample reveals it belongs to the class of trigonal NASICON structures (space group R-3c), with the well-known lantern-like polyhedral interconnection. The Na ions are located on the Na1 sites (6b) and lead to the stoichiometry NaFeNb­(PO_4_)_3_; the presence of empty Na2 sites (site 18e), combined with the presence of the Fe­(III) and Nb­(V) centers, represents a favorable starting point for the subsequent reduction/sodiation of the material.

### 3.2 Functional Characterization of NFNP

The NFNP powder has been subsequently tested in a half cell configuration vs Na; results are reported in Figure 5. The operating potential of the NFNP system has been determined through PCGA measurements. The potential vs charge curves are presented in Figure 5a, which have been used to determine the differential capacity curves without a strong overpotential effect (Figure 5b). All five cycles performed, from the first to the fifth, are also presented in Figure S4, both in terms of potential vs stored charge profiles (panel a) and differential capacity profiles (panel b). PCGA is known for ensuring complete sample reactivity thanks to small potential steps and the choice of a low limiting current. It also allows for the calculation of differential capacitance, with results similar to those obtainable with very low constant currents. It is important to remember that the differential capacitance gives results very similar to those of cyclic voltammetry if the cycle rate is low.

**Figure 5:**
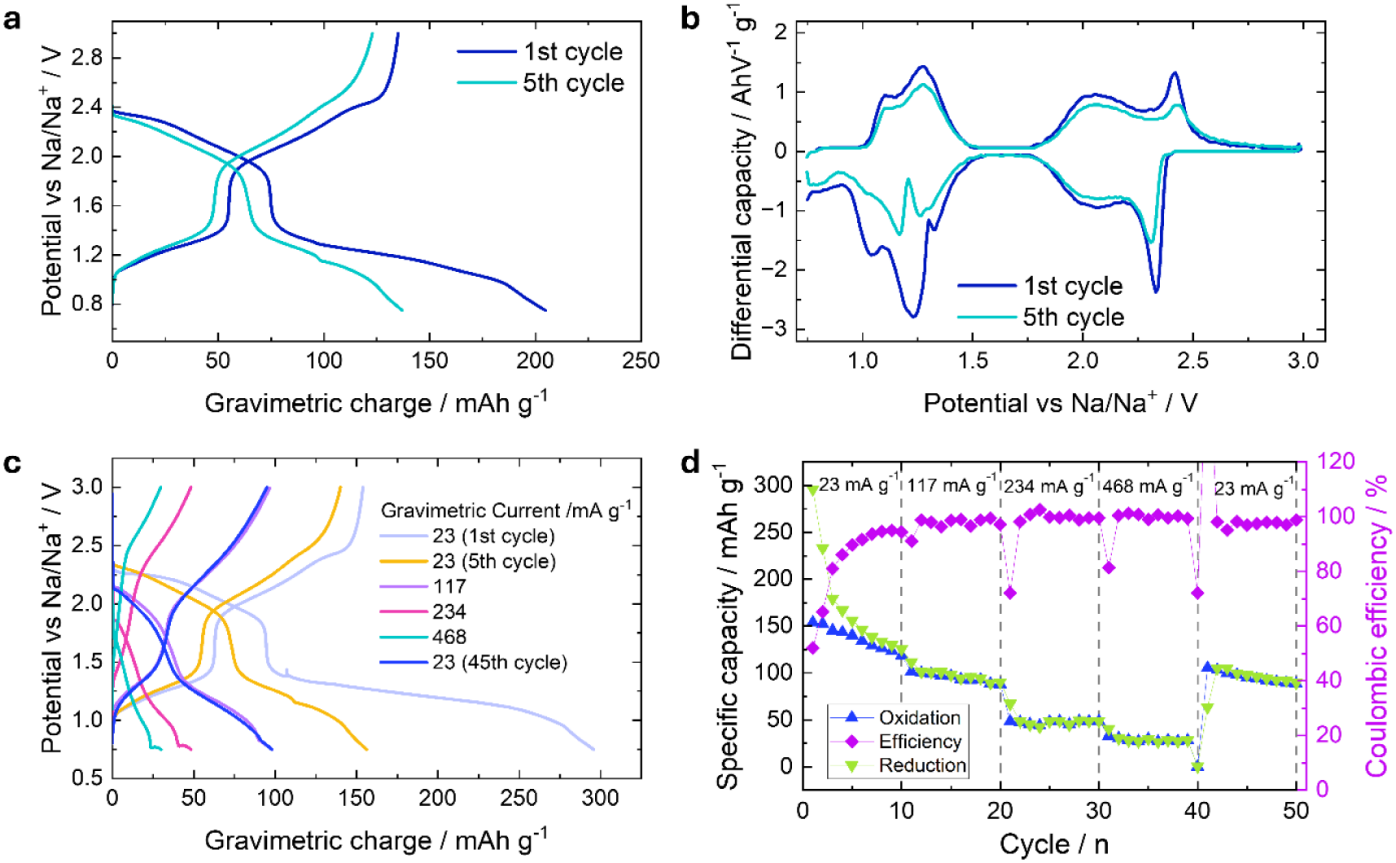
(a) Potential vs charge profiles derived from PCGA analyses; (b) resulting differential capacity for the first and fifth cycles of the NFNP sample; rate test analyses presented as (c) potential vs gravimetric charge profiles; and (d) specific capacity and Coulombic efficiency vs cycle number. The results shown in panels (c) and (d) have been obtained through constant current measurements.

Three main plateaus can be discerned during the first cycle: a sharp plateau above 2.25 V vs Na^+^/Na and two sloping plateaus around 2.00 and 1.20 V vs Na^+^/Na. The obtained profiles and plateau positions are in line with those already reported for the Nb_2_(PO_4_)_3_ material recently proposed as an anode^31,32^ and for the Na_3_Fe_2_(PO_4_)_3_ material, and in general, are associated with the extraction/insertion of Na from different sodium sites within the NASICON framework.^76^ Specifically, these three plateaus can be associated with the reduction/oxidation of Fe­(III)/Fe­(II) (plateau around 2.25 V vs Na^+^/Na), Nb­(V)/Nb­(IV) (around 2.00 V vs Na^+^/Na), and Nb­(IV)/Nb­(III) (around 1.25 V vs Na^+^/Na). To support this hypothesis, the operando XAS data are presented and discussed in more detail in the following section. The first plateau also contains part of the Nb­(V)/Nb­(IV) reaction, with a potential in line with what has been observed in other works.^31,32^ When compared to a subsequent cycle (e.g., the fifth, presented in Figure 5a), the plateaus feature a shortening in the capacity values, which can be attributed to irreversible capacity losses. This is particularly evident in the lowest plateau. The theoretical capacity of this material, based on the reversible insertion/extraction of 3 Na^+^ ions (corresponding to the fully sodiated composition Na_4_FeNb­(PO_4_)_3_), is 176.07 mAh g^– 1^, a value reached and exceeded only during the first sodiation and never retained reversibly. In fact, the reversible capacity of the following cycles, which is around 140–150 mAh g^– 1^, would result in the reversible insertion/extraction of ca. 2.5–2.7 equivalents of Na^+^. The discrepancy in capacity between the first sodiation and desodiation is probably filled both by the consumption of part of the electrolyte due to the formation of the SEI, and by the insertion of the remaining 0.3–0.5 sodium equivalents, which, however, become inactive. This is supported by previous reports, where similar behavior has been observed for other Nb-based materials, and more generally for NASICON materials.^31,77^ The electrochemical insertion processes can be better observed in the differential capacity profiles shown in Figure 5b. In particular, such a plot clearly highlights that, with the disappearance of the irreversible capacity loss contribution to the lowest plateau (here translated into peaks), the material shows a split into two distinct potentials (1.16 and 1.26 V vs Na^+^/Na in reduction). Such a split becomes more evident upon cycling, suggesting an irreversible phase transition of the NASICON structure, which will be discussed in the operando XRD and XAS sections. The electrochemical performance of NFNP has also been evaluated via rate tests, reported in Figure 5c-d through constant current cycling. The potential vs gravimetric charge profiles at low currents provide the same information already described by the aforementioned PCGA measurement: two insertion/extraction steps that can be associated with Nb reduction/oxidation, plus a flat plateau, here clearly observable only in the first cycle, at high potential and associated with Fe reduction (see the operando XAS section). Such profiles remain almost symmetric until high currents (>234 mA g^–1^) are reached. After rate test, when current is reduced again to 23 mA g^–1^, NFNP shows a stable capacity of about 100 mAh g^–1^, corresponding to 1.7 equiv of sodium reversibly inserted and extracted, and a Coulombic efficiency of about 98%.

Moreover, a constant capacity fading is registered, particularly at low gravimetric currents—a trend shared by many NASICONs when used as electrodes,^78^ and which can be caused by the progressive transformation of the material into an inert phase (more details in the following sections).

To better understand the origin of such a discrepancy between the expected and observed specific capacity values and overall electrochemical performance, operando XRD and XAS investigations have been implemented and supported through DFT calculations with the aim of identifying structural relaxation and potential phase transitions, as well as probing the electrochemically active redox couples.

The structural evolution of the materials has been monitored through the acquisition of XRD data, exploiting an optical cell during the first cycle, collected at 15 mA g^–1^ between 0.75 and 3 V vs Na^+^/Na. Results are listed in Figure 6. The evolution of the structure can be followed and related to the charge–discharge profile (Figure 6a-b). With the first plateau, previously associated with the Fe­(III)/Fe­(II) couple, the system undergoes a phase transition with the formation of a secondary phase (see Figure 6a-b for the profile and patterns evolution, selected reflections in Figure 6c-d, and the full operando data set in Figure S6) that becomes more and more evident with the potential scanning. This phase is compatible with the structure recently reported as the product of the sodiation of the Nb_2_(PO_4_)_3_ system, a P-1 phase with the final composition Na_3_Nb_2_(PO_4_)_3_ as supported by profile matching analysis of the profiles for the sodiated compositions.^31^

**Figure 6:**
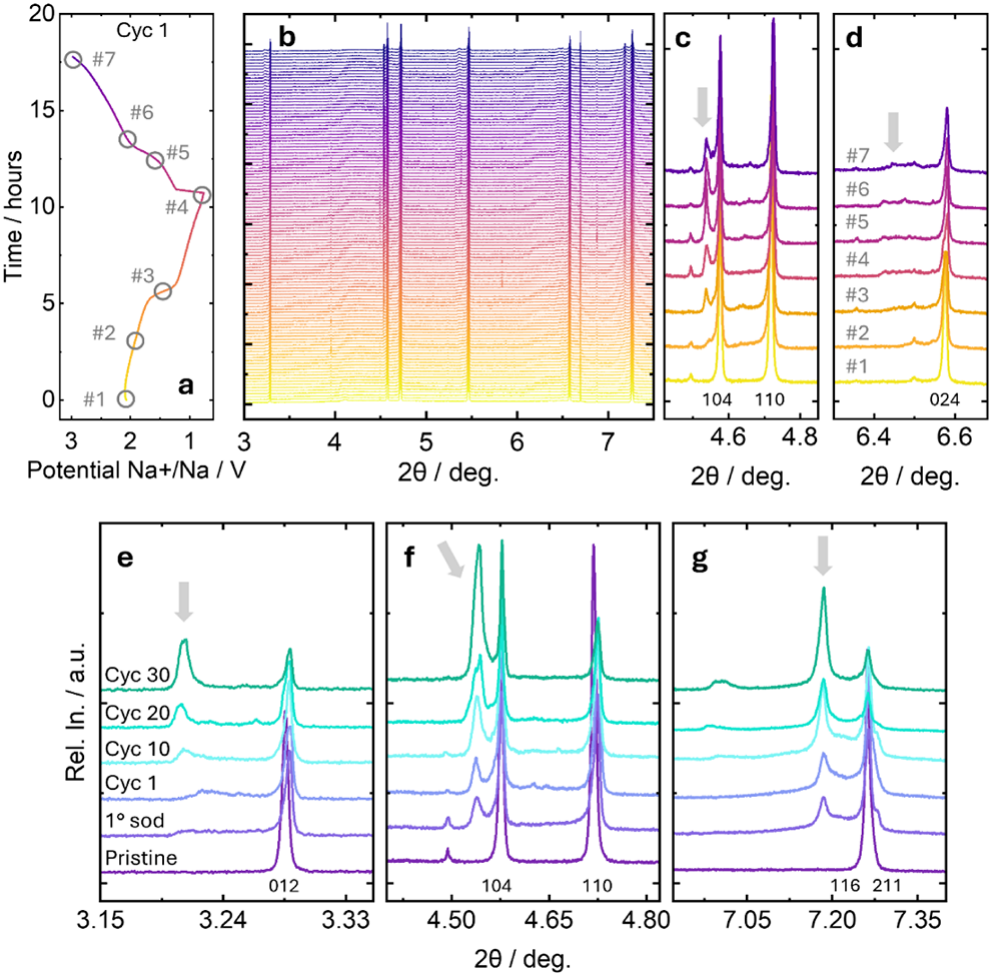
Operando XRD analysis on the NFNP sample cycled vs sodium: (a) first cycle charge–discharge profile of the operando acquisition; (b) corresponding XRD patterns collected during the first cycle; (c, d) details of selected diffraction patterns at different angular ranges for the points 1–7 specified on the charge–discharge profiles; (e, f, g) evolution of the NFNP XRD patterns with subsequent cycling, in particular collected at the OCV, fully sodiated (1°sod), fully desodiated at the end of the first cycle (Cyc 1), and at the OCV after 10 (Cyc 10), 20 (Cyc 20), and 30 (Cyc 30) cycles in different angular ranges.

Moreover, as the Na content increases, the band gap decreases due to more free carriers, which can populate higher energy states or influence the electronic structure. As expected, the Na content also has an influence on the oxidation states of Fe and Nb, as reported in panel (b) of Figure 4 and Figure S5.

During the first sodiation process (Fe­(III) → Fe­(II), between points #1 and #2 in Figure 6a), the reflection associated with the R-3c structure seems to be relatively stable in terms of position (reflections highlighted in Figure 6c and d, patterns #1 and #2). This is compatible with the hypothesis that the original trigonal NFNP systems undergo only a small insertion of sodium up to Na_2_FeNb­(PO_4_)_3_ composition. Further sodium insertion involves the activation of Nb redox couples and triggers a phase transition to the triclinic phase that remains stable throughout the entire reduction process (see arrows in Figure 6c and d). With the subsequent NFNP oxidation, it is not possible to fully recover the pristine structure and composition, as evident from the reflections of the secondary phase still visible after the charge (Figure 6c,d, patterns #6 and #7). From the analysis of the delivered capacity, it is possible to estimate the Na_4_FeNb­(PO_4_)_3_ composition at the end of the reduction, while Na_1.3_FeNb­(PO_4_)_3_ is the estimated composition at the end of the first cycle. The hypothesis that the material is completely sodiated at the end of the reduction arises from the fact that the effective capacity always exceeds the theoretical one in the first cycle (176.07 mAh g^– 1^), which is generally sufficient to admit the reduction to Na_4_FeNb­(PO_4_)_3_ together with the formation of the SEI. From the structural point of view, it is evident from Figure 6c-d that the original R-3c structure is not fully recovered at the end of the first cycle, and a mixture of the two phases is still present. The cell parameters of this phase have been determined as *a* = 8.7222(6), *b* = 9.0921(3), *c* = 22.5422(8), α= 88.932(5), β = 90.231(5), γ = 119.943(3), which are similar to the distortion reported for the sodiated Na_3_Nb_2_(PO_4_)_3_.^31^ Full structural investigation is prevented by the absence of further structural details and the impossibility of isolating the sodiated NFNP compound.

Although this phase is still poorly described, it must be stressed that the pristine NFNP systems do not decompose into known compounds like Na_3_PO_4_ or Nb_*x*_(PO_4_)_γ_, and the phase transition is not inherently harmful to electrochemical performance, as the second phase does still provide similar capacity. However, this newly formed phase could present different conductive properties with respect to the R-3c. Combined with typical issues from phase transitions—such as particle breakage, detachment from the electrode, and mechanical changes affecting the effectiveness of the carbon coating—this may lead to electrode degradation and a reduced fraction of electrochemically active material. Based on this, the phase transition is recognized as the possible cause of the capacity fading with cycles. To assess this aspect, the cell has been further cycled outside the diffractometer, and XRD patterns have been collected in situ at specific points, i.e., at the end of 10, 20, and 30 cycles (Figure 6 e-g). The triclinic phase is formed cycle after cycle at the expense of the trigonal phase, and concomitantly, the capacity decreased from 90 mAh g^–1^ to 70 mAh g^–1^. The fading of the capacity can be associated with the different theoretical capacity of the two phases, their different conductivity, and also the contribution of the mechanical degradation of the electrode due to the phase transition cannot be excluded.

Polymorphs and phase transitions have been widely and extensively investigated for the NASICON family of compounds.^6^ The majority of the NASICON compositions can be described with the R-3c symmetry, and the most commonly reported transition involves the change to C2/c space group, driven by the A content with respect to the AM­(TO_4_)_3_ formula, i.e., the occupancy of the 6b and 18e sites of the mobile species A (generally Li and Na).^6^ Other phase transitions have been reported (P-3, C-1, R-3̅).^6^ It must be noted that the NASICON compounds were originally proposed as cathode materials, with a vast inventory of Na- and Li-rich compositions defined within the R-3c symmetry. These compounds are then depleted of A elements, and the R-3c → C2/c transition is associated with the lower Na and Li content. Moreover, most of the NASICON-based materials considered as electrodes do not show this phase transition evolution during cycling; the R-3c phase is retained and undergoes “breathing” with cell parameters variation.^13,79–81^ In this case, the starting material contains a low amount of Na, as it has been designed as an anode. The structure is thus initially described by R-3c symmetry, but in this case, it is subsequently filled with extra Na; a different structural rearrangement is not surprising. Other Nb-based NASICON compositions have been reported in the literature as possible anode materials, and while some compositions present retention of the crystal structure during cycling,^33,36^ others exhibit a phase evolution associated with the fading of electrochemical performance.^32,82^ A clear correlation between the initial composition (specifically on the M/M’ sites) and the phase evolution is still missing.

To gain a deeper understanding of the electronic and structural modifications occurring in NFNP, as well as the associated charge/discharge mechanisms, operando XANES spectra were recorded at the Nb and Fe K-edges on a working electrochemical cell, as reported in Figure 7. For simplicity, only the potential vs charge profile of the cell used during the Nb-edge analysis is reported in the figure, as it closely matches that of the cell used for the Fe-edge analysis (Figure S7a). The operando experiment is validated by the comparison of ex situ data obtained on electrodes recovered from coin cells and data collected during the second sodiation (Figure S7b-c). The coherence among all the data fully supports the reliability of the cell exploited for the experiment.

**Figure 7:**
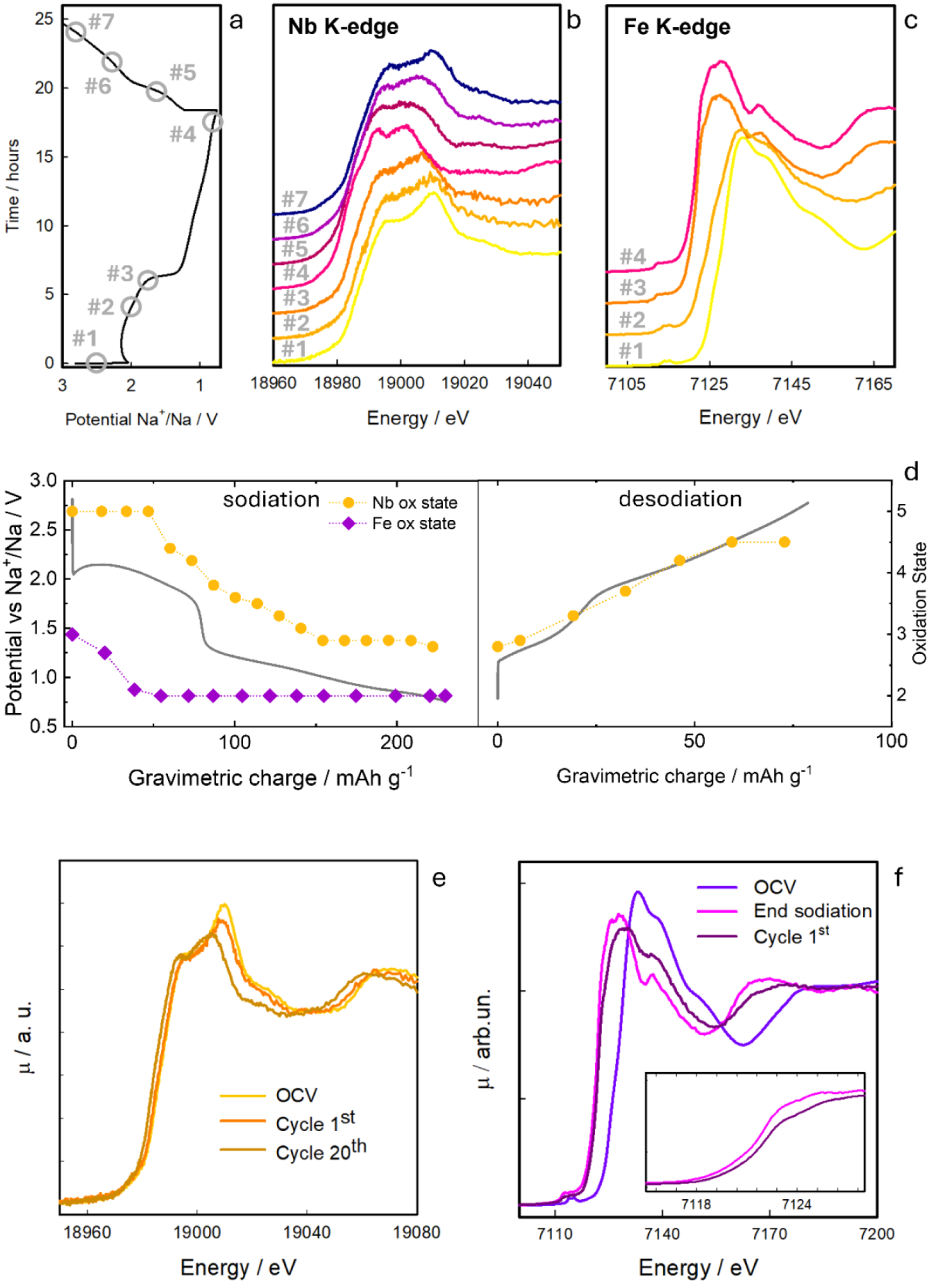
Operando XAS spectra of the NFNP sample cycled vs sodium: (a) first cycle charge–discharge profile of the operando acquisition; corresponding XAS data (b) the Nb K-edge and (c) Fe K-edge at significant potential points of the potential profile (points in panel a); (d) the voltage profile recorded during the first cycle compared to the evolution of the oxidation state of Nb (red circle) and Fe (green squares) as a function of potential; (e) ex situ XAS for selected cycles at the Nb K-edge; and (f) ex situ XAS at the Fe K-edge after one electrochemical cycle, compared with the OCV spectrum and with the spectrum collected at the end of the first sodiation process (0.7 V vs Na^+^/Na). A magnified view of the XANES edge region is shown in the inset.

The evolution of the XAS spectra was monitored, focusing on the changes in the edge energy position and spectral profile during the first sodiation cycle. For clarity, Figure 7b and c display only the spectra acquired at key potentials (points in Figure 7a) at the Nb and Fe K-edges during charge and discharge, respectively, while the complete set of XAS spectra collected during the first cycle is shown in Figure S8. Using the edge energy values from reference samples at the Nb and Fe K-edges (shown in Figure 4c,d), calibration curves were constructed for each edge, allowing determination of the Nb and Fe oxidation states in NFNP throughout the sodiation process. Further details on the determination of the oxidation states can be found in the Supporting Information (Figure S9 and comment). The resulting data, plotted in Figure 7, show the variation in oxidation states of Nb (red circles) and Fe (green squares) as a function of potential alongside the sodiation/desodiation profile (black line). Notably, Fe undergoes a prompt reduction from Fe­(III) to Fe­(II), evident from the shift of the Fe K-edge to lower energies between open-circuit voltage (OCV) and 1.8 V vs Na^+^/Na. Below this potential, no further modifications are observed in the XAS spectrum, either in the edge energy or spectral shape. Within the potential range of 2.5 to 2.0 V vs Na^+^/Na, the Fe^3+^/Fe^2+^ couple is the only active electrochemical system. Indeed, at 2 V vs Na^+^/Na, the Nb K-edge spectrum shows no shift in the edge energy relative to that of the OCV, indicating that Nb remains in the Nb­(V) oxidation state. However, a slight spectral change is observed around 19010 eV, where the spectrum at 2 V vs Na^+^/Na exhibits a reduction in the spectral weight. This change can reflect Na^+^ insertion-induced adjustments in Nb–O bond lengths or coordination environment, which aligns with the small structural changes detected by XRD analysis.

Only below 2 V vs Na^+^/Na, when Fe is fully reduced to Fe­(II), does the reduction of Nb­(V) to Nb­(IV) start to occur, completing at approximately 1.8 V vs Na^+^/Na. Upon further sodiation, particularly between 1.8 and 1.0 V, niobium is progressively reduced to Nb­(III). The spectrum at the end of the sodiation cycle (Figure 7b) not only shows a red shift relative to the OCV spectrum but also reveals significant changes in the spectral shape, indicating substantial modifications in both the electronic and local structure around Nb. These changes are consistent with the occurrence of a phase transition, as already pointed out by the XRD analysis. A similar spectral profile was observed for the Nb_2_(PO_4_)_3_ anode after complete sodiation, where the triclinic phase was also identified.^31^ During desodiation, the spectra exhibit a progressive shift to higher energies, indicating reoxidation of niobium (Figure 7b). Simultaneously, the spectral shape undergoes significant changes, indicating that part of the rhombohedral phase is restored at the expense of the lower symmetry phase, as a consequence of sodium deinsertion. Overall, the process demonstrates a degree of reversibility at the end of the first cycle, although the initial configuration is not completely restored, in nice agreement with operando XRD results. Indeed, by comparing the spectrum obtained at 2.9 V vs Na^+^/Na with the pristine spectrum at OCV, it can be noted that the spectral shape is not exactly coincident, suggesting that the rhombohedral phase is not fully restored. Moreover, the mean oxidation state of Nb is slightly lower than Nb­(V) at the end of the desodiation. To gain additional information on this aspect, ex situ XAS spectra at the Nb K-edge were acquired on electrodes quenched at the OCV after one and 20 electrochemical cycles and compared to the pristine OCV (Figure 7e). It can be observed that the reversibility drastically decreases upon cycling. The spectrum at the 20th cycle shows, indeed, that the in situ formed phase, rich in sodium, is now present in large amounts. This is associated with a reduction of the mean Nb oxidation state, which can be estimated to be ca. 3.7. Concerning the Fe K-edge, it can be seen from Figure 7c and f that, already after one electrochemical cycle, the initial situation is not restored. In particular, as is evident from the spectrum shown in Figure 7f, the mean oxidation state after one cycle is found to be ca. 2.2, suggesting that iron is never completely reoxidized. This is also suggested by the mean potential of the highest potential peak over the cycles (Figure S4c) obtained from the PCGA data, which strongly decreases from cycle one onward, hinting that the component of the peak related to Fe^III^/Fe^II^ reduction is not recovered.

The simulated average voltage (Figure S10) per inserted Na over the entire 1 ≤ *x* ≤ 4 concentration range for Na_*x*_NbFe­(PO_4_)_3_ has been examined, and the results are in agreement with the experimental findings. The examined NASICON system covers a theoretical average voltage range from ∼4.78 V to ∼0.81 V vs Na^+^/Na. The highest intercalation voltage (∼4.78 V vs Na^+^/Na) is computed for NaNbFe­(PO_4_)_3_ for which the Fe oxidation state is (III) and (V) for Nb, in agreement with the experimental results. Upon further insertion of Na into Na_2_NbFe (PO_4_)_3_, Nb remains Nb­(V), but Fe reduces to Fe­(II), contributing to a voltage plateau of ∼2.03 V vs Na^+^/Na. For Na_3_NbFe­(PO_4_)_3_ and Na_4_NbFe­(PO_4_)_3_, the Nb redox couple dominates the intercalation process, with a final oxidation state of Nb^3+^ for Na_4_NbFe­(PO_4_)_3_. These distinct redox transitions result in stepped voltage profiles, highlighting the interplay between the redox chemistry of Fe and Nb during sodium intercalation.

## 4 Conclusion

The NaFeNb­(PO_4_)_3_ compound has been successfully synthesized and characterized from the point of view of its structure and transport properties. It belongs to the class of NASICON compounds, presenting favorable characteristics for its exploitation as an anode for Na-ion batteries, i.e., the presence of empty and accessible sites for extra Na up to Na_4_FeNb­(PO_4_)_3_ compositions combined with the presence of Fe­(II) and Nb­(V) exploitable for reduction, as demonstrated by the diffraction, XAS, and computational investigation. Nevertheless, the electrochemical testing of such materials vs Na demonstrates a fading in performance, as highlighted in Figure 8. This evolution has been deeply investigated through the implementation of in situ XRD and XAS investigations, confirming that a phase evolution is associated with the sodiation of the pristine compound. This phase evolution, leading to low-symmetry NASICON polymorphs, is characterized by being partially irreversible, with the low-symmetry phase accumulating cycle after cycle and being associated with capacity fading, as the Fe­(II/III) couple is not active during the oxidation, again probably associated with the low-symmetry phase, as summarized in Figure 8.

**Figure 8:**
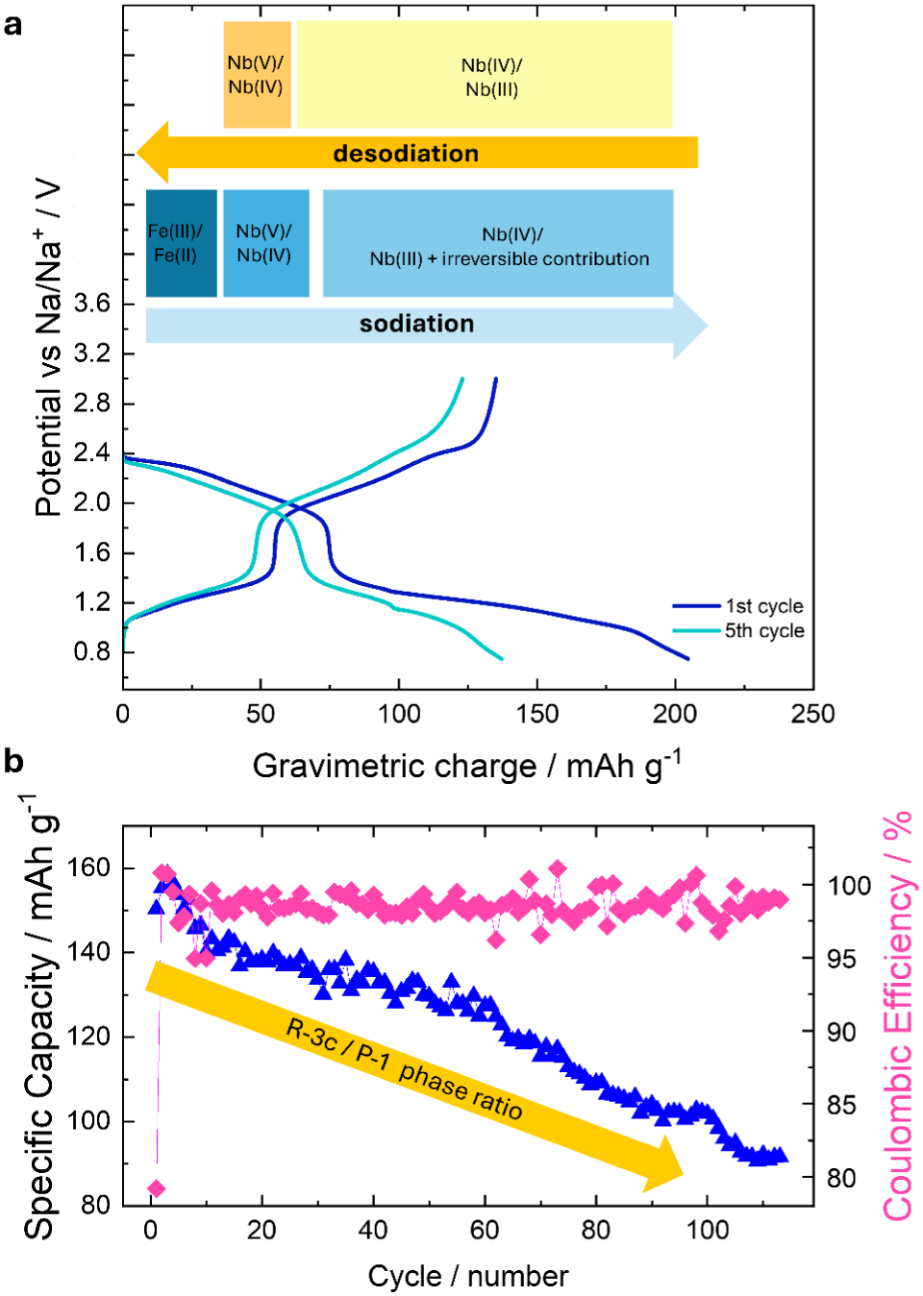
(a) Representation of the electrochemically active species associated with the charge/discharge profiles; and (b) evolution of the specific capacity delivered from NFNP as a function of cycles and the trend of the phase compositions.

## Supplementary Material



## Notes

Refined structural data from XRD and neutron refinements, SEM-EDX data for the NFNP composition, XRD, SEM, TGA data for the uncoated and coated samples, structural data from neutron refinements in the 25–700 °C temperature range, p-DOS for the sodiated compositions, calculated structural parameters for the sodiated compositions, and operando XAS profiles at the Nb–K and Fe–K edges (PDF)

## Abbreviations

NFNP: NaFeNb­(PO_4_)_3_ composition

## References

[1] Goodenough J. B., Hong H. Y.-P., Kafalas J. A. (1976). Fast Na^+^-Ion Transport
in Skeleton Structures. Mater. Res. Bull..

[2] Hong H. Y.-P. (1978). Crystal
Structure and Ionic Conductivity of Li_14_Zn­(GeO_4_)_4_ and Other New Li^+^ Superionic Conductors. Mater. Res. Bull..

[3] Yin J.-H., Zhu H., Yu S.-J., Dong Y.-B., Wei Q.-Y., Xu G.-Q., Xiong Y., Qian Y. (2023). Recent Advances of LATP and Their
NASICON Structure as a Solid-State Electrolyte for Lithium-Ion Batteries. Adv. Eng. Mater..

[4] Luo C., Yi M., Cao Z., Hui W., Wang Y. (2024). Review of Ionic Conductivity
Properties of NASICON Type Inorganic Solid Electrolyte LATP. ACS Appl. Electron. Mater..

[5] Singh B., Wang Z., Park S., Gautam G. S., Chotard J.-N., Croguennec L., Carlier D., Cheetham A. K., Masquelier C., Canepa P. (2021). A Chemical Map of NaSICON Electrode Materials for Sodium-Ion
Batteries. J. Mater. Chem. A.

[6] Avdeev M. (2021). Crystal Chemistry
of NaSICONs: Ideal Framework, Distortion, and Connection to Properties. Chem. Mater..

[7] Zhang N., Di H., Wen B., Luo J., Zhang L., Zhang S. (2024). Doping Enhanced
Charge Transportation in NASICONs for Energy Storage. Nano Energy.

[8] Xiao J., Zhang B., Liu J., He X., Xiao Z., Qin H., Liu T., Amine K., Ou X. (2024). NaSICON-Type Materials
for Lithium-Ion Battery Applications: Progress and Challenges. Nano Energy.

[9] Liu Y., Sun C., Li Y., Jin H., Zhao Y. (2023). Recent Progress of
Mn-Based NASICON-Type Sodium Ion Cathodes. Energy
Storage Mater..

[10] Thirupathi R., Kumari V., Chakrabarty S., Omar S. (2023). Recent Progress and
Prospects of NASICON Framework Electrodes for Na-Ion Batteries. Prog. Mater. Sci..

[11] Rajagopalan R., Zhang Z., Tang Y., Jia C., Ji X., Wang H. (2021). Understanding Crystal Structures, Ion Diffusion Mechanisms and Sodium
Storage Behaviors of NASICON Materials. Energy
Storage Mater..

[12] Chen S., Wu C., Shen L., Zhu C., Huang Y., Xi K., Maier J., Yu Y. (2017). Challenges
and Perspectives for NASICON-Type
Electrode Materials for Advanced Sodium-Ion Batteries. Adv. Mater..

[13] Hou J., Chen T., Ait Tamerd M., Liu J., Huang W.-H., Hadouchi M., Zhu Y., Shi X., Guo S., Yang M., Shi Y., Tang M., Ma J. (2025). Activating
Fast and Reversible Sodium Storage in NASICON Cationic Defect Sites
through Fluorine Doping. Nat. Commun..

[14] Zhu Y., Xu H., Bao Y., Li S., Chen Y. (2025). Ternary NASICON-Type
Na _3.25_VMn_0.25_Fe_0.75_(PO_4_)_3_/NC@CNTs Cathode with Reversible Multielectron Reaction
and Long Life for Na-Ion Batteries. ACS Appl.
Mater. Interfaces.

[15] Puspitasari D. A., Patra J., Hernandha R. F. H., Chiang Y.-S., Inoishi A., Chang B. K., Lee T.-C., Chang J.-K. (2024). Enhanced Electrochemical
Performance of Ca-Doped Na_3_V_2_(PO_4_)_2_F_3_/C Cathode Materials for Sodium-Ion Batteries. ACS Appl. Mater. Interfaces.

[16] Liao X., Li Y., Xie B., Xie M., Tan X., Zheng Q., Li L., Zhao X.-X., Gu Z.-Y., Smith S. C., Zhao J., Lin D., Wu X.-L. (2025). Unlocking Advanced Sodium Storage Performance: High-Entropy
Modulates Crystallographic Sites with Reversible Multi-Electron Reaction. Energy Storage Mater..

[17] Chen Y., Peng P., Sun K., Wu L., Zheng J. (2024). Stabilizing
NASICON-Type Na_4_MnCr­(PO_4_)_3_ by Ti-Substitution
toward a High-Voltage Cathode Material for Sodium Ion Batteries. J. Colloid Interface Sci..

[18] Sharma A., Anand T. S., Urkude R., Gupta A., Ganguli A. K. (2024). Na_3_V_2_(PO_4_)_3_/C in Symmetric Cells:
Evaluating Anode and Cathode Performance. J.
Phys. Chem. C.

[19] Zhang X., Wu Y., Mei D., Wen S., Zhang H. (2024). NASICON-Na­(NbO_2_)_2_PO_4_ Anode Material with High Capacity
and Good Stability for Sodium-Ion Batteries. Ionics.

[20] Cherkashchenko I. R., Panin R. V., Dembitskiy A. D., Novichkov D. A., Aksyonov D. A., Antipov E. V., Khasanova N. R. (2024). Reversible
Multielectron Redox Activity of the Anti-NASICON-Type Phosphate LiNbV­(PO_4_)_3_ towards Lithium and Sodium Intercalation. Dalton Trans..

[21] Panin R. V., Cherkashchenko I. R., Zaitseva V. V., Samigullin R. R., Zakharkin M. V., Novichkov D. A., Babkin A. V., Mikheev I. V., Khasanova N. R., Antipov E. V. (2024). Realizing Three-Electron Redox Reactions
in NASICON-Type NaCrNb­(PO_4_)_3_ for Sodium Ion
Battery Applications. Chem. Mater..

[22] Wang D., Liu Q., Chen C., Li M., Meng X., Bie X., Wei Y., Huang Y., Du F., Wang C., Chen G. (2016). NASICON-Structured
NaTi_2_(PO_4_)_3_@C Nanocomposite as the
Low Operation-Voltage Anode Material for High-Performance Sodium-Ion
Batteries. ACS Appl. Mater. Interfaces.

[23] Man Y., Sun J., Zhao X., Duan L., Fei Y., Bao J., Mo X., Zhou X. (2023). An Ultrastable Sodium-Ion Battery Anode Enabled by
Carbon-Coated Porous NaTi_2_(PO_4_)_3_ Olive-like
Nanospheres. J. Colloid Interface Sci..

[24] Mukherjee A., Akhtar M., Chang J.-K., Banerjee S., Majumder S. B. (2023). Reduced
Graphene Oxide and Carbon Nanotube Anchored NASICON-Type NaTi_2_(PO_4_)_3_ Nanocomposite Anodes for High-Rate
Performance Sodium-Ion Batteries. Mater. Chem.
Phys..

[25] Wang Q., He S., Chen H., Peng Z., Xu Z., Zeng Z., Wang C., Xue P., Ni L., Li X., Han J. (2024). Tailoring the Electronic
Structure of the NaTi_2_(PO_4_)_3_ Anode
for High-Performing Sodium-Ion Batteries
via Defect Engineering. Green Chem..

[26] He B., Yin K., Gong W., Xiong Y., Zhang Q., Yang J., Wang Z., Wang Z., Chen M., Man P., Coquet P., Yao Y., Sun L., Wei L. (2021). NaTi_2_(PO_4_)_3_ Hollow Nanoparticles Encapsulated in
Carbon Nanofibers as Novel Anodes for Flexible Aqueous Rechargeable
Sodium-Ion Batteries. Nano Energy.

[27] Hou Z., Zhang X., Chen J., Qian Y., Chen L., Lee P. S. (2022). Towards High-Performance
Aqueous Sodium Ion Batteries:
Constructing Hollow NaTi_2_(PO_4_)_3_@C
Nanocube Anode with Zn Metal-Induced Pre-Sodiation and Deep Eutectic
Electrolyte. Adv. Energy Mater..

[28] Xu T., Zhao M., Li Z., Su Z., Ren W., Yang S., Pol V. G. (2022). A High Rate and
Long Cycling Performance
NaTi_2_(PO_4_)_3_ Core–Shell Porous
Nanosphere Anode for Aqueous Sodium-Ion Batteries. Energy Technol..

[29] Lv T., Suo L. (2021). Water-in-Salt Widens the Electrochemical Stability Window: Thermodynamic
and Kinetic Factors. Curr. Opin. Electrochem..

[30] Khalid S., Pianta N., Mustarelli P., Ruffo R. (2023). Use of Water-In-Salt
Concentrated Liquid Electrolytes in Electrochemical Energy Storage:
State of the Art and Perspectives. Batteries.

[31] Patra B., Kumar K., Deb D., Ghosh S., Gautam G. S., Senguttuvan P. (2023). Unveiling
a High Capacity Multi-Redox (Nb^5+^/Nb^4+^/Nb^3+^) NASICON-Nb_2_(PO_4_)_3_ Anode
for Li- and Na-Ion Batteries. J. Mater. Chem.
A.

[32] Patra B., Hegde R., Natarajan A., Deb D., Sachdeva D., Ravishankar N., Kumar K., Gautam G. S., Senguttuvan P. (2024). Stabilizing
Multi-Electron NASICON-Na_1.5_V_0.5_Nb_1.5_(PO_4_)_3_ Anode via Structural Modulation for
Long-Life Sodium-Ion Batteries. Adv. Energy
Mater..

[33] Patra B., Narayanan S., Halder S., Sharma M., Sachdeva D., Ravishankar N., Pati S. K., Jain S. K., Senguttuvan P. (2025). NASICON-NaV_0.25_Al_0.25_Nb_1.5_(PO_4_)_3_/C: A High-Rate and Robust Anode for Fast Charging and Long-Life
Sodium-Ion Batteries. Adv. Mater..

[34] Cherkashchenko I. R., Panin R. V., Babkin A. V., Novichkov D. A., Antipov E. V., Khasanova N. R. (2025). NaAlNb­(PO_4_)_3_ NASICON-Type Phosphate with the Nb^5+^/Nb^4+^/Nb^3+^ Multielectron Redox Activity on
Sodium Intercalation. Mendeleev Commun..

[35] Panin R. V., Cherkashchenko I. R., Novichkov D. A., Mikheev I. V., Stolbov D. N., Antipov E. V., Khasanova N. R. (2025). New NASICON-Type Phosphates, KNbM­(PO_4_)_3_ (M = Ti, V), Exhibiting a Reversible Alkali
Metal Intercalation. Inorg. Chem. Commun..

[36] Khasanova N. R., Panin R. V., Cherkashchenko I. R., Zakharkin M. V., Novichkov D. A., Antipov E. V. (2023). NaNbV­(PO_4_)_3_: Multielectron NASICON-Type Anode Material for Na-Ion
Batteries
with Excellent Rate Capability. ACS Appl. Mater.
Interfaces.

[37] Chen X., Chen K., Ji F., Han L., Ai X., Cao Y., Fang Y. (2025). Achieving Fast Ion/Electron Transportation and Smooth
Phase Transition in Polyanion Cathode by the High Entropy Strategy. Adv. Energy Mater..

[38] Rodríguez-Carvajal J. (1993). Recent Advances
in Magnetic Structure Determination by Neutron Powder Diffraction. Phys. B.

[39] Fischer P., Frey G., Koch M., Könnecke M., Pomjakushin V., Schefer J., Thut R., Schlumpf N., Bürge R., Greuter U., Bondt S., Berruyer E. (2000). High-Resolution
Powder Diffractometer HRPT for Thermal Neutrons at SINQ. Phys. B.

[40] Ghoshal S., Jia Q., Bates M. K., Li J., Xu C., Gath K., Yang J., Waldecker J., Che H., Liang W., Meng G., Ma Z.-F., Mukerjee S. (2017). Tuning Nb–Pt
Interactions To Facilitate Fuel Cell Electrocatalysis. ACS Catal..

[41] Ravel B., Newville M. (2005). ATHENA , ARTEMIS ,
HEPHAESTUS: data analysis for X-ray
absorption spectroscopy using IFEFFIT. J. Synchrotron
Radiat..

[42] Fitch A., Dejoie C., Covacci E., Confalonieri G., Grendal O., Claustre L., Guillou P., Kieffer J., de Nolf W., Petitdemange S., Ruat M., Watier Y. (2023). ID22 –
the High-Resolution Powder-Diffraction Beamline at ESRF. J. Synchrotron Radiat..

[43] Kresse G., Hafner J. (1993). *Ab Initio* Molecular Dynamics for Liquid
Metals. Phys. Rev. B.

[44] Kresse G., Hafner J. (1994). *Ab Initio* Molecular-Dynamics Simulation
of the Liquid-Metal–Amorphous-Semiconductor Transition in Germanium. Phys. Rev. B.

[45] Kresse G., Furthmüller J. (1996). Efficiency
of Ab-Initio Total Energy Calculations for
Metals and Semiconductors Using a Plane-Wave Basis Set. Comput. Mater. Sci..

[46] Perdew J. P., Burke K., Ernzerhof M. (1996). Generalized
Gradient Approximation
Made Simple. Phys. Rev. Lett..

[47] Blöchl P. E. (1994). Projector
Augmented-Wave Method. Phys. Rev. B.

[48] Grimme S., Antony J., Ehrlich S., Krieg H. (2010). A Consistent and Accurate *Ab Initio* Parametrization of Density Functional Dispersion
Correction (DFT-D) for the 94 Elements H-Pu. J. Chem. Phys..

[49] Liechtenstein A. I., Anisimov V. I., Zaanen J. (1995). Density-Functional Theory and Strong
Interactions: Orbital Ordering in Mott-Hubbard Insulators. Phys. Rev. B.

[50] Dudarev S. L., Botton G. A., Savrasov S. Y., Humphreys C. J., Sutton A. P. (1998). Electron-Energy-Loss Spectra and
the Structural Stability
of Nickel Oxide: An LSDA+U Study. Phys. Rev.
B.

[51] Wang L., Maxisch T., Ceder G. (2006). Oxidation Energies
of Transition
Metal Oxides within the GGA + U Framework. Phys.
Rev. B.

[52] Lee Y.-L., Kleis J., Rossmeisl J., Morgan D. (2009). Ab Initio Energetics
of LaBO_3_ (001) (B = Mn, Fe, Co, and Ni) for Solid Oxide
Fuel Cell Cathodes. Phys. Rev. B.

[53] Monkhorst H. J., Pack J. D. (1976). Special Points for
Brillouin-Zone Integrations. Phys. Rev. B.

[54] Henkelman G., Uberuaga B. P., Jónsson H. (2000). A Climbing Image Nudged Elastic Band
Method for Finding Saddle Points and Minimum Energy Paths. J. Chem. Phys..

[55] Ahsan M. T., Ali Z., Usman M., Hou Y. (2022). Unfolding the Structural Features
of NASICON Materials for Sodium-ion Full Cells. Carbon Energy.

[56] Huang Q., Chen G., Zheng P., Li W., Wu T. (2021). NASICON-Structured
Na Ion Conductor for next Generation Energy Storage. Funct. Mater. Lett..

[57] Zatovsky I. V., Ushchapivska T. I., Slobodyanik N. S., Ogorodnyk I. V. (2006). Structure
and Polymorphism of Complex Phosphates Prepared from Fluxes of the
Na_2_O-P_2_O_5_-Fe_2_O_3_-Nb_2_O_5_ System. Russ.
J. Inorg. Chem..

[58] Berry F. J., Vithal M. (1995). New Compounds with NASICON-Related
Structures of the
Type NaM′M″P_3_O_12_ (M′ Nb,
Sb; M″ Al, Ga, In, Fe). Polyhedron.

[59] Zhou Q., Wang L., Li W., Zhao K., Liu M., Wu Q., Yang Y., He G., Parkin I. P., Shearing P. R., Brett D. J. L., Zhang J., Sun X. (2021). Sodium Superionic Conductors
(NASICONs) as Cathode Materials for Sodium-Ion Batteries. Electrochem. Energy Rev..

[60] Li H., Xu M., Gao C., Zhang W., Zhang Z., Lai Y., Jiao L. (2020). Highly Efficient,
Fast and Reversible Multi-Electron Reaction of
Na_3_MnTi­(PO_4_)_3_ Cathode for Sodium-Ion
Batteries. Energy Storage Mater..

[61] Boilot J. P., Collin G., Colomban P. (1988). Relation Structure-Fast
Ion Conduction
in the NASICON Solid Solution. J. Solid State
Chem..

[62] Jacobson S. H., Ratner M. A., Nitzan A. (1981). Stoichiometry-Dependent
Conductivity
in Framework Ionic Conductors. Phys. Rev. B.

[63] Helm B., Schlem R., Wankmiller B., Banik A., Gautam A., Ruhl J., Li C., Hansen M. R., Zeier W. G. (2021). Exploring
Aliovalent Substitutions in the Lithium Halide Superionic Conductor
Li_3– x_In_1–x_Zr_x_Cl_6_ (0 ≤ *x* ≤ 0.5). Chem. Mater..

[64] Rosa C., Ravalli M., Pianta N., Mustarelli P., Ferrara C., Quartarone E., Malavasi L., Sheptyakov D., Tealdi C. (2024). Aliovalent Substitution
in Li_3_InCl_6_: A Combined Experimental and Computational
Investigation of Structure
and Ion Diffusion in Lithium-Halide Solid State Electrolytes. ACS Appl. Energy Mater..

[65] Bdey S., Savvin S. N., Bourguiba N. F., Núñez P. S. (2022). Crystal
Structure and Na^+^ Transport in Na_3_La­(AsO_4_)_2_. J. Solid State Chem..

[66] Li C., Li R., Liu K., Si R., Zhang Z., Hu Y. (2022). NaSICON: A
Promising Solid Electrolyte for Solid-state Sodium Batteries. Interdiscip. Mater..

[67] Shimanouchi R., Takatsuka A., Ozeki R., Kawano Y. (2019). Preparation and Electric
Conductivity of Na_3_Al_2_(PO_4_)_3_ Ceramic from a Hydrothermally Synthesized Precursor. Mater. Chem. Phys..

[68] Hwang J.-Y., Myung S.-T., Sun Y.-K. (2017). Sodium-Ion Batteries: Present and
Future. Chem. Soc. Rev..

[69] d’Yvoire F., Pintard-Scrépel M., Bretey E., de la Rochère M. (1983). Phase Transitions
and Ionic Conduction in 3D Skeleton Phosphates A_3_M_2_(PO_4_)_3_: A = Li, Na, Ag, K ; M
= Cr, Fe. Solid State Ionics.

[70] He Y., Scivally E., Shaji A., Ouyang B., Zeng Y. (2025). Unraveling
the Fast Ionic Conduction in NASICON-Type Materials. Adv. Energy Mater..

[71] Wang J., He T., Yang X., Cai Z., Wang Y., Lacivita V., Kim H., Ouyang B., Ceder G. (2023). Design Principles for NASICON Super-Ionic
Conductors. Nat. Commun..

[72] Park H., Jung K., Nezafati M., Kim C.-S., Kang B. (2016). Sodium Ion
Diffusion in Nasicon (Na_3_Zr_2_Si_2_PO_12_) Solid Electrolytes: Effects of Excess Sodium. ACS Appl. Mater. Interfaces.

[73] Zhao X., Zhang Z., Zhang X., Tang B., Xie Z., Zhou Z. (2018). Computational screening
and first-principles investigations of NASICON-type
Li x M 2 (PO 4) 3 as solid electrolytes for Li batteries. J. Mater. Chem. A.

[74] Liu Y., Li J., Shen Q., Zhang J., He P., Qu X., Liu Y. (2022). Advanced Characterizations and Measurements for Sodium-Ion Batteries
with NASICON-Type Cathode Materials. eScience.

[75] Fracchia M., Visibile A., Ahlberg E., Vertova A., Minguzzi A., Ghigna P., Rondinini S. (2018). α- and γ-FeOOH: Stability,
Reversibility, and Nature of the Active Phase under Hydrogen Evolution. ACS Appl. Energy Mater..

[76] Liu Y., Zhou Y., Zhang J., Xia Y., Chen T., Zhang S. (2017). Monoclinic Phase Na_3_Fe_2_(PO_4_)_3_: Synthesis, Structure, and Electrochemical
Performance as
Cathode Material in Sodium-Ion Batteries. ACS
Sustainable Chem. Eng..

[77] Senguttuvan P., Rousse G., Vezin H., Tarascon J.-M., Palacín M. R. (2013). Titanium­(III)
Sulfate as New Negative Electrode for Sodium-Ion Batteries. Chem. Mater..

[78] Jian Z., Hu Y., Ji X., Chen W. (2017). NASICON-Structured Materials for
Energy Storage. Adv. Mater..

[79] Wu X., Li Z., Zhang J., Sun X., Xue R., Zhu H., Ding J., Liu R. (2025). Insight into
Highly Reversible Multielectron
V^3+^/V^4+^/V^5+^ Reaction of High-Entropy
Doped NASICON Cathode for Sodium Ion Batteries. ACS Appl. Mater. Interfaces.

[80] Hou J., Hadouchi M., Sui L., Liu J., Tang M., Kan W. H., Avdeev M., Zhong G., Liao Y.-K., Lai Y.-H., Chu Y.-H., Lin H.-J., Chen C.-T., Hu Z., Huang Y., Ma J. (2021). Unlocking
Fast and Reversible Sodium
Intercalation in NASICON Na_4_MnV­(PO_4_)_3_ by Fluorine Substitution. Energy Storage Mater..

[81] Hou J., Hadouchi M., Sui L., Liu J., Tang M., Hu Z., Lin H., Kuo C., Chen C., Pao C., Huang Y., Ma J. (2023). Insights into
Reversible Sodium Intercalation
in a Novel Sodium-Deficient NASICON-Type Structure: Na_3.40_□_0.60_Co_0.5_Fe_0.5_V­(PO_4_)_3_. Small.

[82] Gonzalez-Correa E., Mazumder M., Kumar K., Ghosh S., Senguttuvan P., Clément R. J. (2025). High Voltage
Irreversibilities in NASICON Na_3+y_V_2–y_Mg_y_(PO_4_)_3_ Cathodes. Chem. Mater..

