# Supplementary material for: NaFeNb(PO4)3 as an Electrode Material for Sodium-Ion Batteries: Insights into Phase Evolution and Capacity Fading
Source: Chem Mater. 2026 Jan 8;38(2):657–71. doi: 10.1021/acs.chemmater.5c01854 (PMC12856997; doi:10.1021/acs.chemmater.5c01854)
Supplement: Supplementary file 1 [file cm5c01854_si_001.pdf]

SUPPLEMENTARY INFORMATION

The $NaFeNb(PO_4)_3$ as Electrode Material for Sodium-Ion Batteries: Insights into Phase Evolution and Capacity Fading.

Nicolò Pianta,[a] Shahid Khalid,[a] Ivan Claudio Pellini, [a] Domenico Antonio Florenzano,[a] Gabriele Brugnetti, [a,b] Nicole Ceribelli,[a] Luca Olivi,[c] Giuliana Aquilanti,[c] Denis Sheptyakov, [d] Andrew Nicholas Fitch, [e] Martina Fracchia,[f,g] Livia Giordano,[a] Riccardo Ruffo,[a,g] Chiara Ferrara, [a,g]*

[a] Department of Materials Science, Università degli Studi di Milano Bicocca, Via Cozzi 55, Milano 20125, Italy

[b] Ricerca sul Sistema Energetico - RSE S.p.A., Via R. Rubattino 54, Milano 20134, Italy

[c] Elettra – Sincrotrone Trieste, Strada Statale Strada Statale 14 - km 163,5 in AREA Science Park 34149 Basovizza, Trieste, Italy

[d] Laboratory for Neutron Scattering and Imaging, Paul Scherrer Institut, 5232 Villigen PSI, Switzerland

[e] European Synchrotron Radiation Facility, 71 Avenue des Martyrs, 38000 Grenoble, France

[f] Department of Chemistry, Università di Pavia, Via T. Taramelli 12, Pavia 27100, Italy

[g] GISEL - INSTM, Consorzio Interuniversitario per la Scienza e Tecnologia dei Materiali, Via G. Giusti 9, 50121 Firenze, Italy

Table S1. Structural parameters from refinement room temperature x ray and neutron data.

			**RT XRD**		
	a / Å	c / Å	V / Å^3	Fraction / w%	Rwp, Chi2
**$NaFeNb(PO_4)_3$**	8.59304(3)	22.09246(8)	1412.760(8)	94.60(76)	22.0, 30.3
**$NbPO_5$**				1.05(9)	
**$NaFeP_2O_7$**				4.35(18)	
			**$NaFeNb(PO_4)_3$**		
**Species**	x	y	z	occ	Biso
**Na1**	0	0	0	0.80	(Baiso)
**Fe/Nb**	0	0	0.14506(6)	0.45/0.56	0.592(32)
**P**	0.28765(3(9	0	0.25	1	0.619(76)
**O1**	0.19480(62)	0.16840(62)	0.08902(22)	1	0.691(84)
**O2**	0.02791(72)	0.20324(67)	0.19395(23)	1	0.691(84)
			**RT Neutron**		
	a / Å	c / Å	V / Å^3	Fraction / w%	Rwp, Chi2
**$NaFeNb(PO_4)_3$**	8.59720(18)	22.07001(4)	1412.691(52)	94.03(79)	4.81, 3.08
**$NbPO_5$**				0.14(21)	
**$NaFeP_2O_7$**				5.84(24)	
			**$NaFeNb(PO_4)_3$**		
**Species**	x	y	z	occ	Biso
**Na1**	0	0	0	0.83	(Baniso)
**Fe/Nb**	0	0	0.14494(8)	0.54/0.46	0.480(5)
**P**	0.28600(25)	0	0.25	1	0.348(12)
**O1**	0.19250(21)	0.16647(22)	0.08872(6)	1	1.101(17)
**O2**	0.02424(22)	0.19978(22)	0.19352(6)	1	1.101(17)

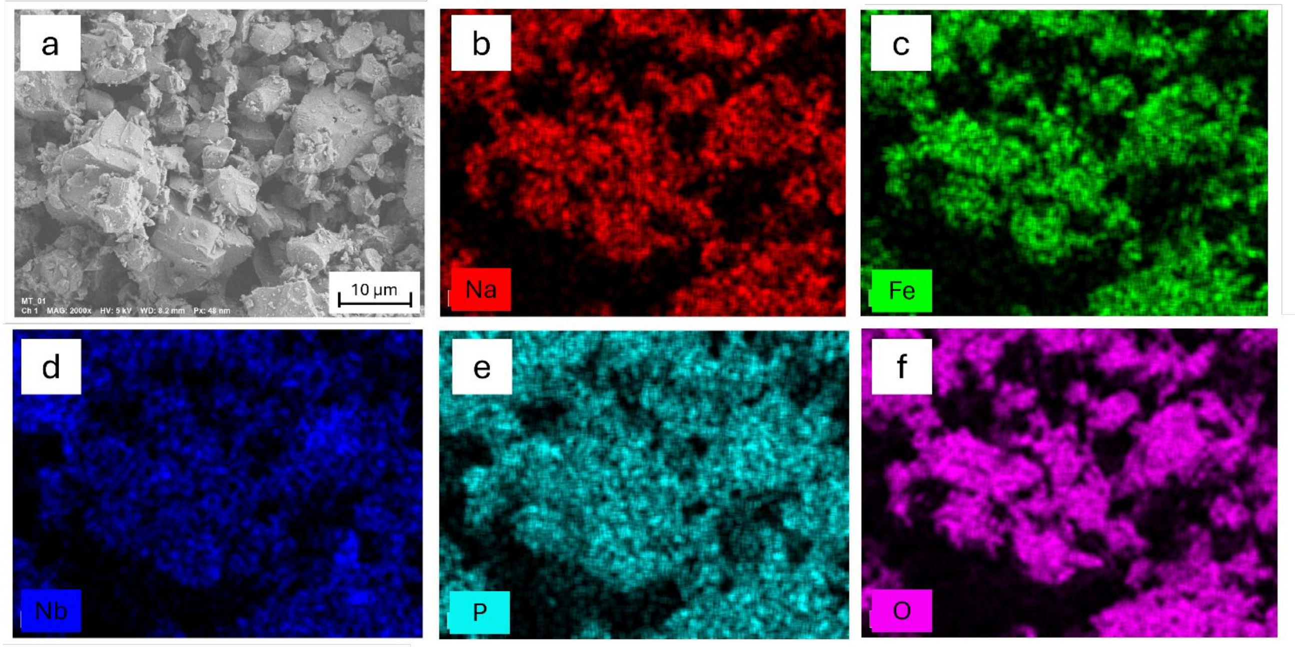

Figure S1. (a) SEM image for the pristine NFNP sample together with EDX maps revealing the distribution of (b) P, (c) Nb, (d) Na, (e) Fe, and (f) O.

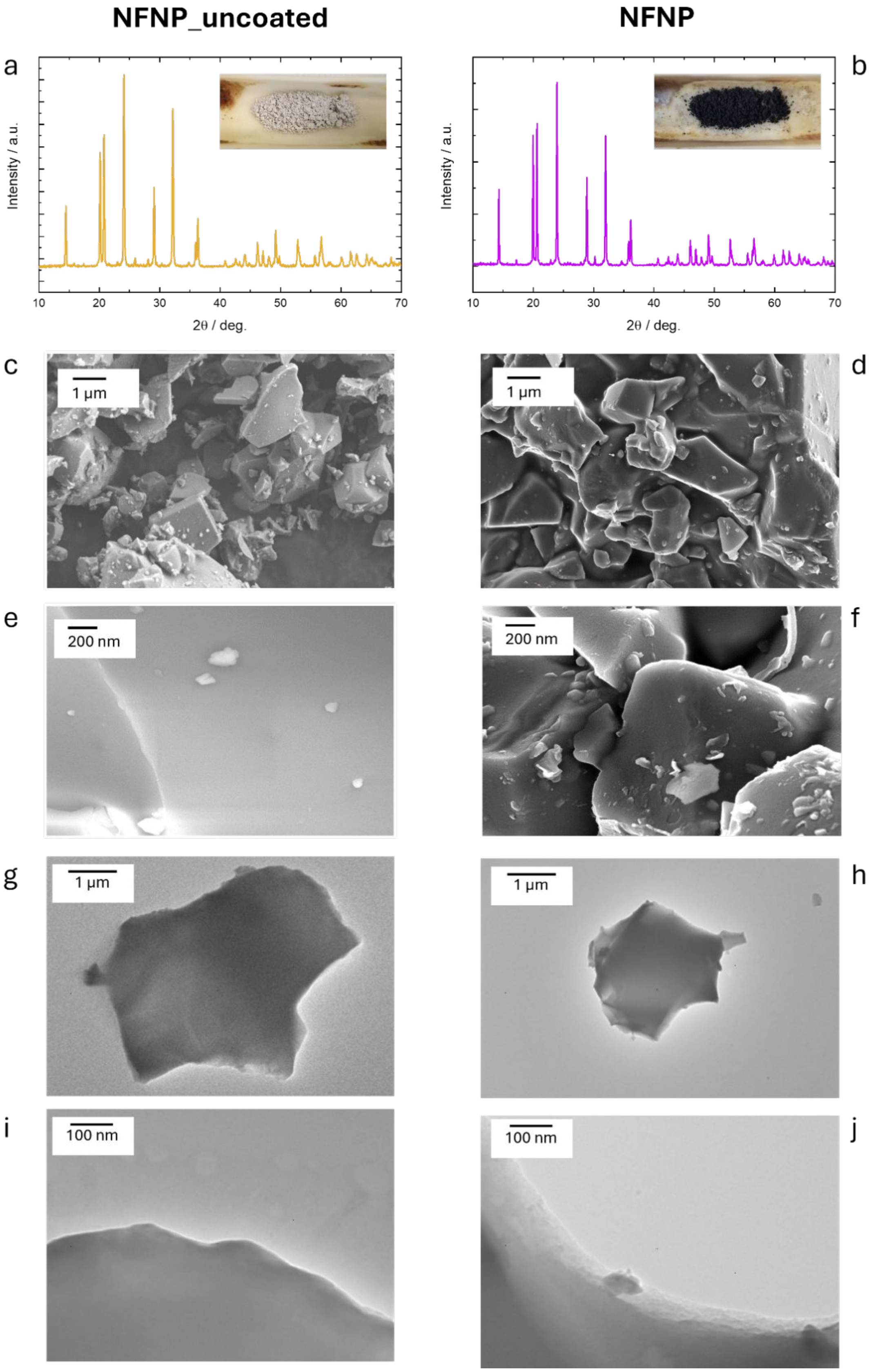

Figure S2. (a,b) XRD, (c, d, e, f) SEM images, (g, h, i, j) TEM images for the NFNP_uncoated and NFNP samples

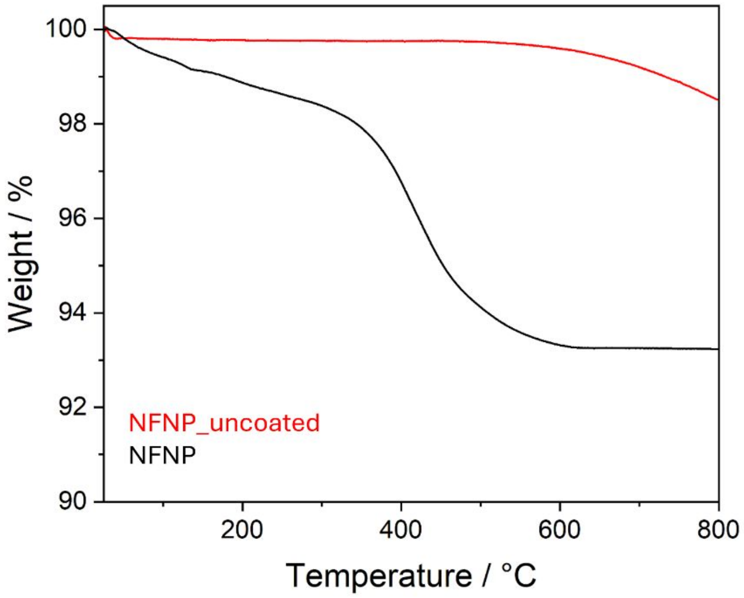

Figure S3. TGA profile for the uncoated and coated NFNP samples.

Table S2. CHNS results for the NFNP_uncoated and NFNP samples.

	C / %	H / %	N / %	S / %
**NFNP_UNCoATED**	0.01	0.000	0.13	0.089
**NFNP**	4.32	0.228	1.31	0.186

Table S3. Results of temperature dependent neutron diffraction data. The Fe/Nb and Na_{tot} values have been refined for the room temperature data and then constrained at that value during the refinements of data at higher temperature as no reactivity is expected from the sample already treated at high temperature.

T / °C	a / Å	c / Å	V / Å³	Na1 / occ	Na2 /occ	Rwp / Chi2
**25**	8.59720(18)	22.07001(4)	1412.691(52)	0.83	-	4.81, 3.08
**300**	8.58532(38)	22.19854(103)	1416.995(90)	0.83	-	6.69, 2.39
**500**	8.57867(4)	22.29485(116)	1420.937(119)	0.83	-	6.23, 3.06
**700**	8.57891(51)	22.38999(149)	1427.080(152)	0.75	0.08	5.97, 3.67

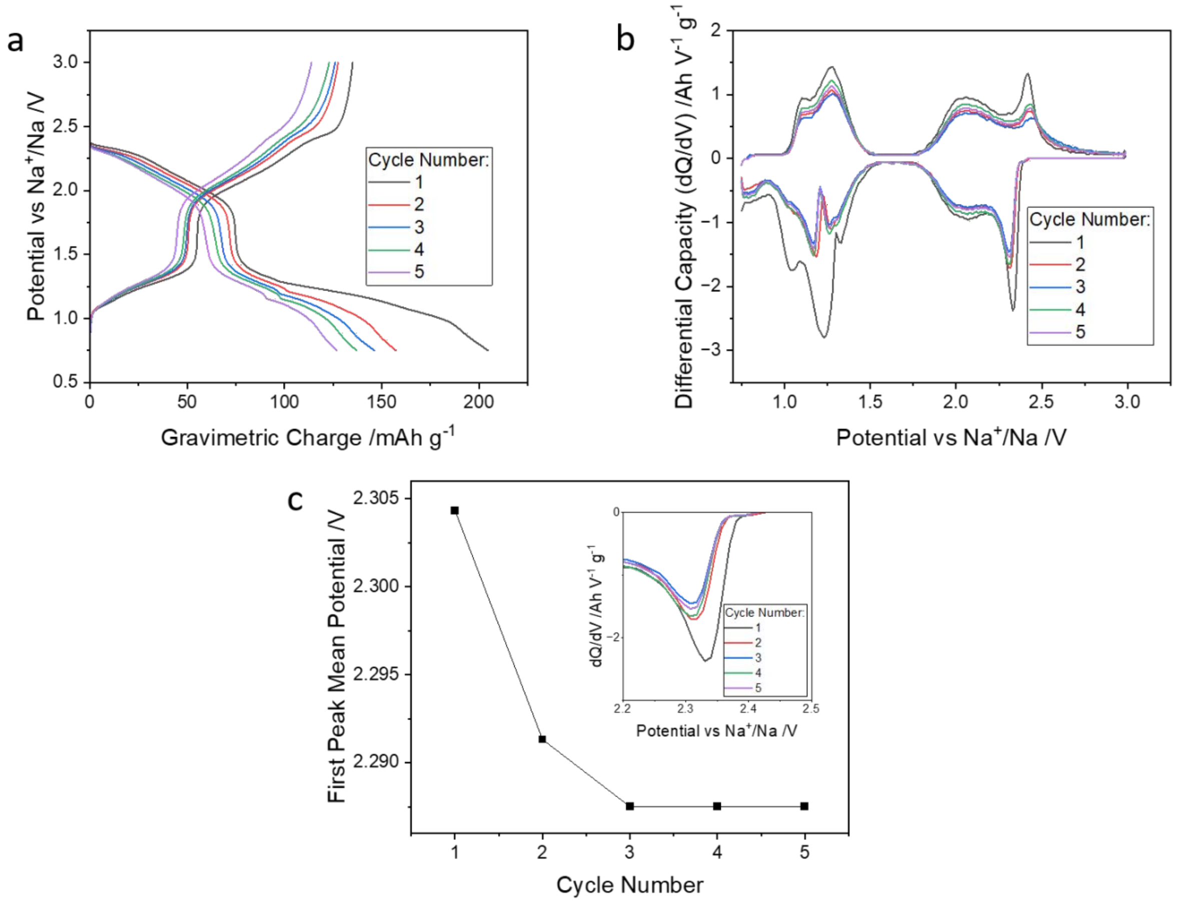

Figure S4. Results of the PCGA analysis. (a) Potential vs charge and (b) differential capacity profiles (from first to fifth cycle), (c) mean potential of the highest reduction peak.

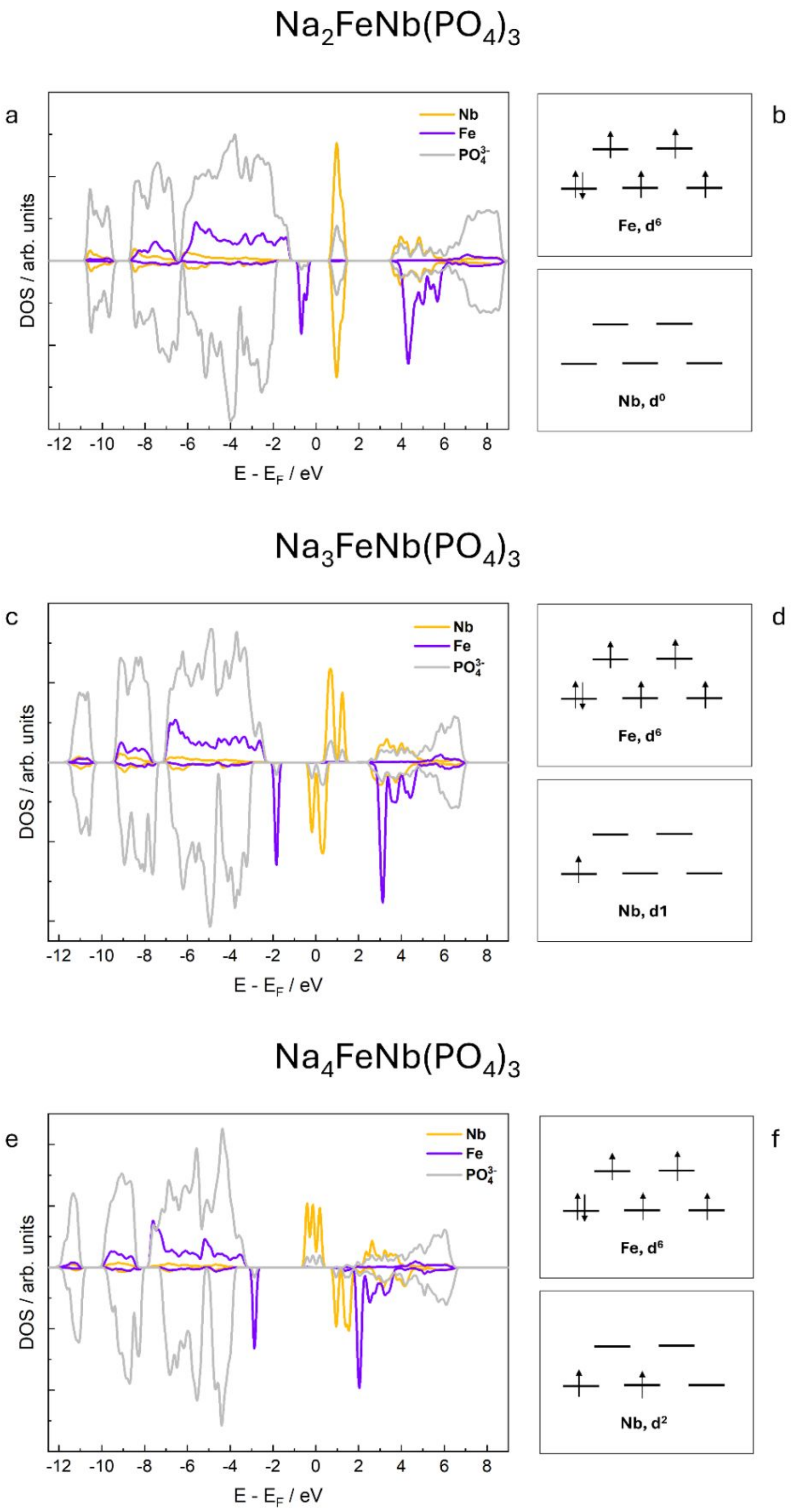

Figure S5. (a, c, e) atom-projected DOS of $Na_xNbFe(PO_4)_3$, (b, d, f) oxidation state with x = 2, 3, 4, following the electrochemical sodiation process.

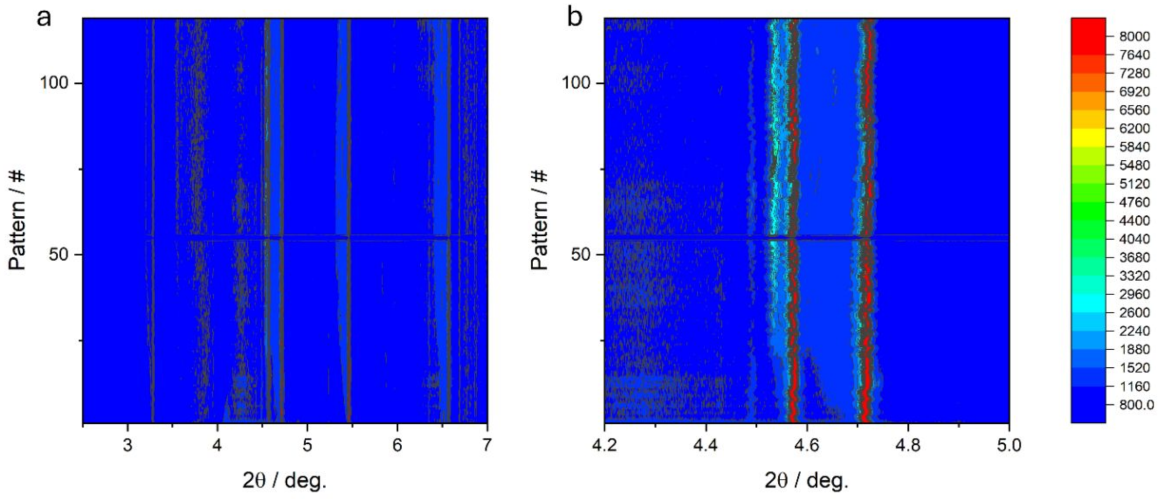

Figure S6. Full operando XRD dataset obtained during the first cycle (reduction / oxidation) for the NFNP sample (a) in the 2.5 – 7 degrees angular range and (b) zoom in the 4.2 – 5.0 degrees angular range highlighting the appearance of new reflection.

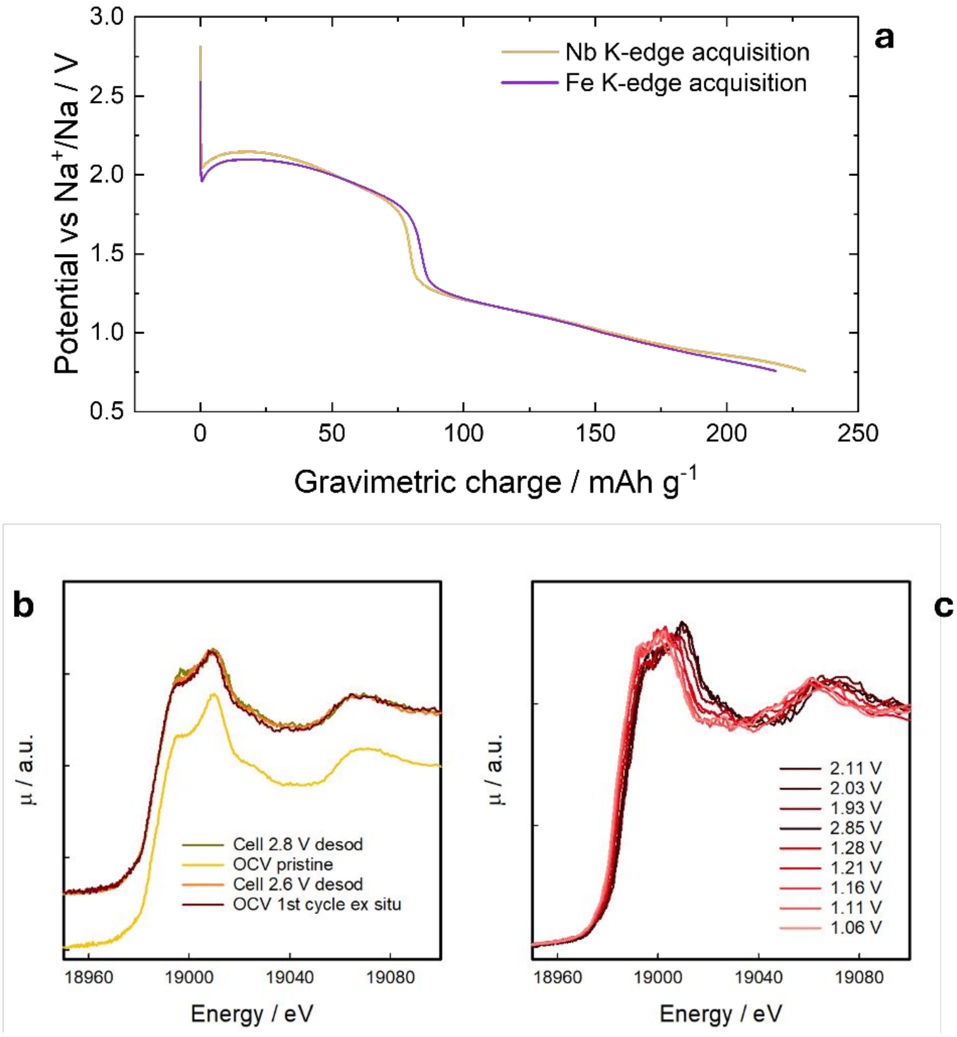

Figure S7. Potential vs gravimetric charge profiles measured during the sodiation of the operando cells used for the operando Nb- (yellow) and Fe- (purple) edges XAS analyses (a), ex situ and in situ spectra collected at the Nb K-edge (b), spectra collected at the Nb K-edge during the second sodiaction (cycle II) (c).

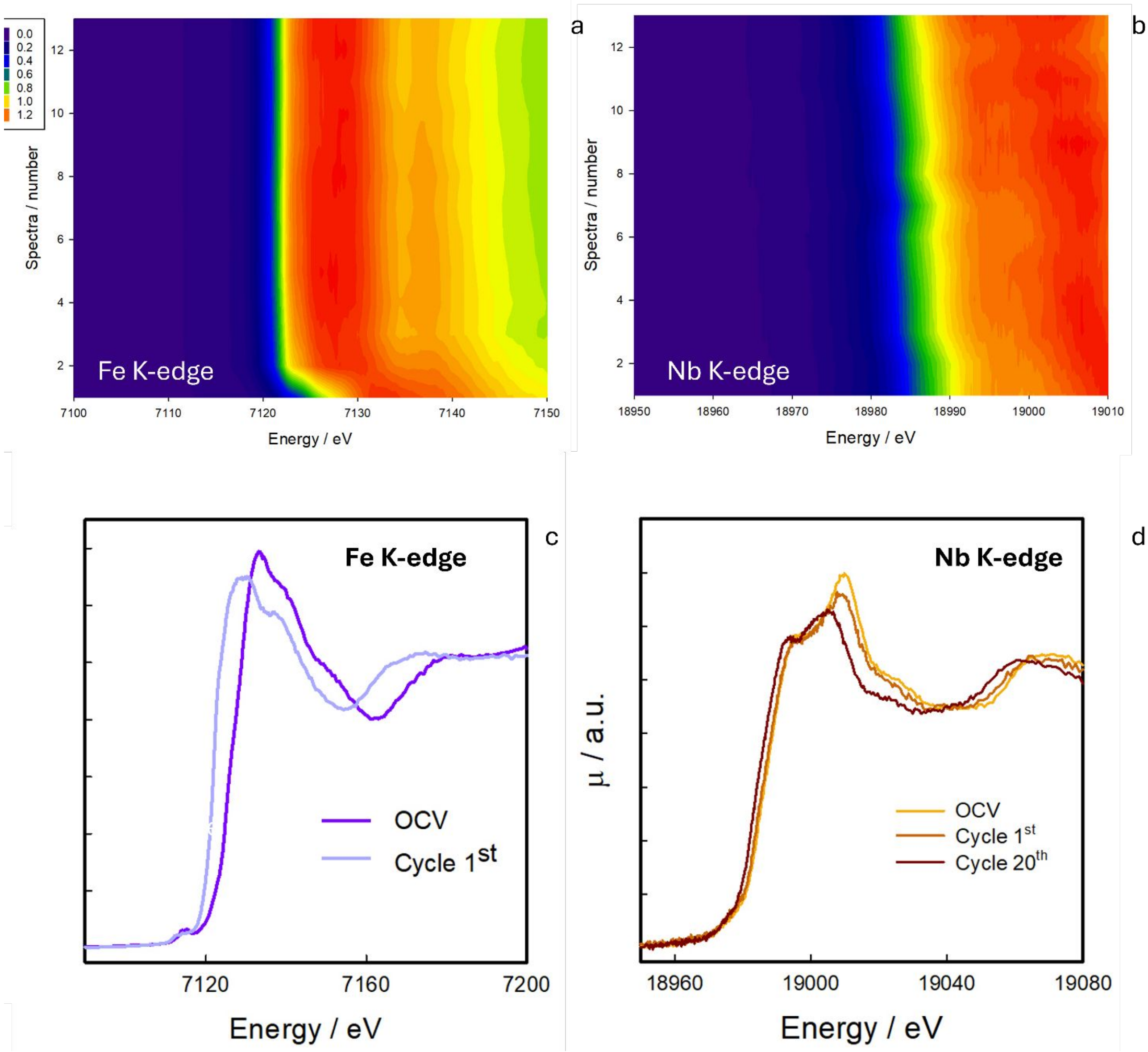

Figure S8. Full operando XAS data set obtained (a) at the Fe K-edge and (b) at the Nb K-edgeand ex situ XAS for selected cycles at (c) Fe K-edge and (d) at the Nb K-edge.

Determination of the Nb and Fe oxidation states

At the Fe K-edge, the oxidation state can be determined straightforwardly by comparison with reference spectra. In particular, spectra of FeOOH and FeO (Figure 4 in the main text) were used as standards, allowing us to assign oxidation states of +3 and +2 to the pristine and fully sodiated electrodes, respectively. The spectra recorded at 2.07 and 1.97 V were well reproduced by a linear combination of the spectra at OCV and at the end of sodiation, and the oxidation states were derived from the fitting coefficients. All other spectra overlapped with that of the fully sodiated electrode.

At the Nb K-edge, the oxidation state was evaluated using a calibration curve obtained from the reference compounds shown in Figure 4. In this case, the determination of the edge energy as the maximum of the first derivative is not straightforward, due to the presence of composition-dependent pre-edge features or shoulders that significantly affect the derivative position. To overcome this issue, the edge energy was instead estimated

as the energy corresponding to an absorption coefficient of 0.6, a region where differences in spectral shape among the standards are not so evident. This procedure has been successfully applied in other systems, for example at the Mn K-edge (E. Céspedes et al., J. Appl. Phys. 2007, 102, 033907) and leads to a reliable calibration curve. Accordingly, the oxidation state at each potential was calculated from the energy value at $\mu = 0.6$ using the calibration curve shown in Figure S8.

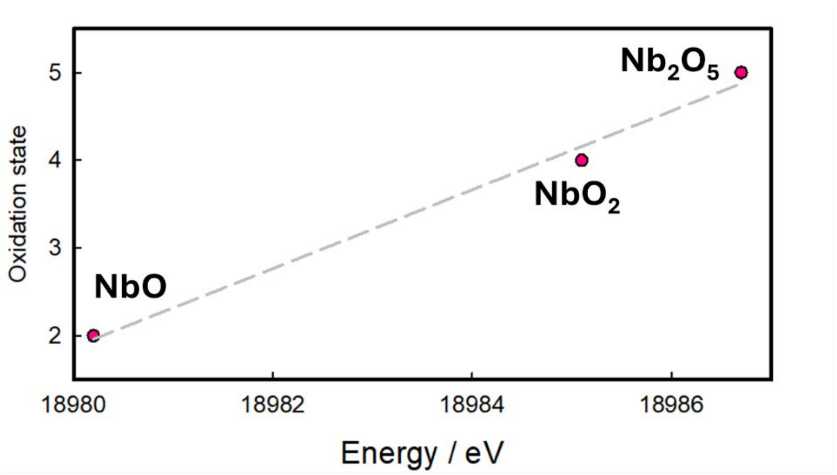

Figure S9: Calibration curve for the determination of the Nb oxidation state.

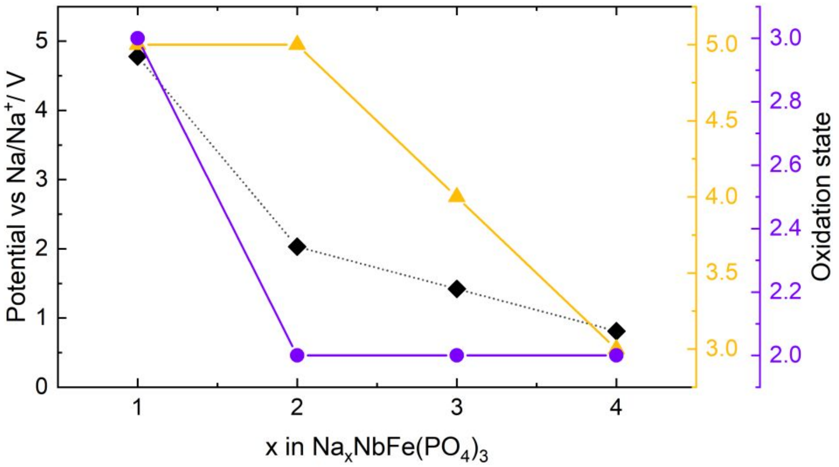

Figure S10. Calculated average voltage for the range $1 \leq x \leq 4$ with respect to the $Na_xFeNb(PO_4)_3$ composition (Nb orange, Fe purple).
